# “Late-onset” ADHD symptoms in young adulthood: is this ADHD?

**DOI:** 10.1177/10870547211066486

**Published:** 2022-01-16

**Authors:** Lucy Riglin, Robyn E Wootton, Lucy A Livingston, Jessica Agnew-Blais, Louise Arseneault, Rachel Blakey, Sharifah Shameem Agha, Kate Langley, Stephan Collishaw, Michael C O’Donovan, George Davey Smith, Evie Stergiakouli, Kate Tilling, Anita Thapar

**Affiliations:** 1 Division of Psychological Medicine and Clinical Neurosciences, MRC Centre for Neuropsychiatric Genetics and Genomics, Cardiff University, UK; 2 Population Health Sciences and MRC Integrative Epidemiology Unit, University of Bristol, Bristol, UK; 3 Nic Waals Institute, Lovisenberg Diaconal Hospital, Oslo, Norway; 4 School of Psychology, Cardiff University, Wales, UK; 5 Social, Genetic, and Developmental Psychiatry Centre, Institute of Psychiatry, Psychology and Neuroscience, King's College London, UK; 6 Department of Psychology, School of Biological and Chemical Sciences, Queen Mary University London, UK; 7 Cwm Taf Morgannwg University Health Board, Wales, UK

**Keywords:** ALSPAC, ADHD, late-onset, longitudinal, genetic, scaffolding, compensation

## Abstract

**Objective:**

We investigated whether “late-onset” ADHD that emerges in adolescence/adulthood is similar in risk factor profile to: 1) child-onset ADHD, but emerges later because of scaffolding/compensation from childhood resources; and 2) depression, because it typically onsets in adolescence/adulthood and shows symptom and genetic overlaps with ADHD.

**Methods:**

We examined associations between late-onset ADHD and ADHD risk factors, cognitive tasks, childhood resources and depression risk factors in a population-based cohort follow-up to age 25 years (N=4224-9764).

**Results:**

Parent-rated late-onset ADHD was like child-onset persistent ADHD in associations with ADHD polygenic risk scores and cognitive task performance, although self-rated late-onset ADHD was not. Late-onset ADHD was associated with higher levels of childhood resources than child-onset ADHD and did not show strong evidence of association with depression risk factors.

**Conclusions:**

Late-onset ADHD shares characteristics with child-onset ADHD when parent-rated, but differences for self-reports require investigation. Childhood resources may delay the onset of ADHD.

The DSM-5 currently conceptualises ADHD as a neurodevelopmental disorder that onsets in childhood, prior to age 12 years (American Psychiatric Association, 2013). However, several studies suggest that ADHD symptoms can first emerge during adolescence or adult life in some individuals (Asherson & Agnew-Blais, 2019), although these findings are controversial (Faraone & Biederman, 2016) and it is unclear whether ADHD that has an apparent onset after 12 years of age, so called late-onset ADHD, is similar to ADHD that onsets in childhood. One possibility is that they are fundamentally the same disorders with the same underlying pathophysiology, but that for some individuals the clinical features are compensated for, or otherwise obscured, in childhood. Another possibility is that the two are fundamentally different disorders. For example, it is possible that late-onset ADHD might represent a variant of a later-onset disorder such as depression.

Findings that late-onset ADHD is associated with higher cognitive ability than child-onset ADHD (Breda et al., 2021; Kosaka, Fujioka, & Jung, 2019) could support the “compensation” hypothesis for late-onset ADHD; that is, where some children have an underlying liability to ADHD but may possess characteristics (internal resources) that allow them to compensate for this liability. Other characteristics that could compensate for ADHD liability have yet to be examined, nor has research investigated external resources (e.g. family resources) that might “scaffold” underlying ADHD liability in childhood and thus delay symptom onset (Asherson & Agnew-Blais, 2019). However, current evidence suggests that late-onset ADHD is not associated with an increased burden of ADHD risk alleles (common variants) (Agnew-Blais et al., 2021; Manfro et al., 2019; Moffitt et al., 2015; Riglin et al., 2016), which raises the possibility that when late-onset ADHD occurs, it is a manifestation of a different disorder. Depression typically first arises at the same age as late-onset ADHD (i.e. adolescence and early adulthood) (Thapar, Collishaw, Pine, & Thapar, 2012). Moreover, there is some overlap between the symptoms of depression and ADHD, including inattention and restlessness, which means that questionnaire measures of ADHD might detect depression symptomatology. Also, although ADHD shows strong genetic overlap with many psychiatric disorders; the strongest genetic correlation is with depression (Demontis et al., 2019) and late-onset ADHD, like depression, appears to be more common in females (Asherson & Agnew-Blais, 2019). Thus, an alternative hypothesis is that late-onset ADHD is actually a variant of depression. Research examining the hypothesis that late-onset ADHD is a manifestation of other psychopathology has typically strictly excluded those meeting diagnostic criteria for these disorders from the late-onset group (Agnew-Blais et al., 2016; Caye et al., 2016). However, excluding individuals with a comorbid disorder from research into late-onset ADHD diagnosis is overly simplistic, given high comorbidity between ADHD and depression (Asherson & Agnew-Blais, 2019; Fischer et al., 2007; Spencer, Biederman, & Wilens, 1999). Another approach to investigating whether late-onset ADHD is a form of ADHD or depression is to determine whether late-onset ADHD shares risk factors with ADHD or depression.

Interpreting findings on late-onset ADHD is complicated by methodological issues. One important consideration is who reports the symptoms. In childhood, ADHD symptoms are usually reported by a parent, but this typically changes to self-report after the age of 18 years. Agreement between parent- and self-reported ADHD symptoms is typically low. In clinical studies, affected individuals even in adult life tend to under-report symptoms (e.g. Barkley, Fischer, Smallish, & Fletcher, 2002) compared with other informants, whereas in population studies, individuals tend to self-report more symptoms (e.g. Riglin et al., 2020). Given low agreement between parent- and self-reports, different individuals may be identified by different informants (Asherson & Agnew-Blais, 2019) and it is not currently clear if these capture a similar underlying psychopathology.

A second methodological consideration is how late-onset ADHD is operationalised. Most longitudinal studies have defined late-onset ADHD in individuals who surpass validated thresholds for ADHD symptom counts (Agnew-Blais et al., 2021; Agnew-Blais et al., 2016; Caye et al., 2016; Cooper et al., 2018; Manfro et al., 2019; Moffitt et al., 2015; Sibley et al., 2018; Taylor, Larsson, Gillberg, Lichtenstein, & Lundström, 2019). An alternative data-driven approach is to group individuals into latent trajectory classes according to observed patterns of ADHD symptoms across development. To our knowledge only one study has used this approach to investigate associations with late-onset ADHD (Breda et al., 2021), finding associations with female sex and higher IQ, but did not investigate associations with genetic risk or cognitive tasks. Finally, different measures of ADHD may capture different individuals and potentially different psychopathology: for example, screening questionnaires that include only a few ADHD items may identify a less ADHD-specific phenotype compared to measures which assess all 18 DSM ADHD symptoms.

Our study aimed to investigate the nature of late-onset ADHD, utilising different informants (parent, self), operationalisations (observed cut-points, latent-trajectories) and measures (Strengths and Difficulties Questionnaire screening questionnaire, Development and Well-Being Assessment 18 DSM ADHD symptoms). We tested two hypotheses: (1) late-onset symptoms reflect a similar disorder to child-onset ADHD, but with delayed symptom onset. If this hypothesis is correct, we would predict that young-adults with late-onset and child-onset ADHD symptoms will show similar ADHD risk factor profiles and cognitive impairments, but that those with late-onset would have higher levels of childhood internal and external resources that may allow compensation for underlying ADHD liability or obscure the presence of symptoms in childhood (i.e. “compensation” or “scaffolding”). (2) late-onset ADHD symptoms reflect a variant of depression: in this case, compared to those with child-onset ADHD symptoms, young-adults with late-onset symptoms will show stronger associations with depression risk factors.

## Methods

### Sample

We analysed data from the Avon Longitudinal Study of Parents and Children (ALSPAC), a well-established prospective birth cohort study. Pregnant women resident in Avon, UK with expected dates of delivery 1st April 1991 to 31st December 1992 were invited to take part in the study. When the oldest children were approximately 7 years of age, an attempt was made to bolster the initial sample with eligible cases who had failed to join the study originally, resulting in a total of 14,901 study offspring alive at 1 year of age. Where families included multiple births, we included the oldest sibling. Full details of this study are provided in the Supplementary Material.

### ADHD

ADHD symptoms were measured using the 5-item ADHD subscale of the Strengths and Difficulties Questionnaire (SDQ)(Goodman, 1997) and the 18-item Development and Well-Being Assessment (DAWBA) ADHD section (Goodman, Ford, Richards, Gatward, & Meltzer, 2000). The SDQ is a brief screening questionnaire and was completed by parents about their children at approximately ages 4, 7, 8, 9, 12, 13, 17 and 25 years and additionally by self-report at age 25 years. Continuous SDQ ADHD scores (possible range 0-10) at ages 4-17 years can be categorised as low (0-5), slightly raised (6-7) or high (8-10)(Goodman, 1997) whereas at age 25 years the recommended cut-point for high symptoms is ≥4 for parent-reports and ≥5 for self-reports (Riglin, Agha, 2021). The DAWBA is a structured diagnostic interview that assesses the 18 DSM ADHD diagnostic symptoms and was completed by parents as a questionnaire at approximately 7, 10, 13, 15 and 25 years and used to generate symptom scores (possible range 0-36). Lifetime ADHD medication use (methylphenidate, dexamfetamine or atomoxetine) was assessed by selfreport at age 25 years.

### ADHD risk factors

#### ADHD genetic risk

Genetic risk for ADHD was indexed using polygenic risk scores (PRS). PRS were generated using PRSice version 1.25 (Euesden, Lewis, & O’Reilly, 2015) based on GWAS of ADHD (Demontis et al., 2019). Genotyping details as well as full methods for generating the PRS are presented in the Supplementary Material.

#### Perinatal risk factors

Preterm birth (<37 weeks gestation) and low birth weight (<2500g) were included as risk factors for ADHD (Thapar, Cooper, Eyre, & Langley, 2013) for singletons (99% of the primary sample).

### Cognitive tasks

We investigated cognitive tasks that index characteristic features of ADHD: attention and response inhibition.

#### Sustained attention

Sustained attention was assessed using the Tests of Everyday Attention for Children (TEA-Ch)(Robertson, Ward, Ridgeway, & Nimmo-Smith, 1996) Sky Search task at age 8 years and the Sustained Attention Task (SART)(Bellgrove, Hawi, Kirley, Gill, & Robertson, 2005) at age 25 years. More detail on these measures are given in the Supplementary Material. Sustained attention scores were multiplied by minus one so that higher scores reflect better cognitive performance and subsequently standardized to mean=0 SD=1 to aid interpretation.

#### Response inhibition

Inhibitory control was assessed using the TEA-Ch Opposite Worlds task, which is a type of Stroop task (Stroop, 1935) at age 8 years and the Double Trouble task (Metzler-Baddeley, Caeyenberghs, Foley, & Jones, 2016) at age 25 years. More detail on these measures are given in the Supplementary Material. Child response inhibition scores were multiplied by minus one so that both child and young-adult higher scores reflect better cognitive performance; scores were subsequently standardized to mean=0 SD=1 to aid interpretation.

### Childhood resources

We examined internal and external resources in childhood that may compensate for, or scaffold symptoms. These included (i) verbal IQ, given evidence that this may facilitate compensation in relation to autistic behaviours (Livingston, Colvert, Bolton, & Happé, 2019; Livingston & Happé, 2017), (ii) childhood reading ability, given that higher scholastic performance may delay the detection of autism symptoms (Livingston, Shah, & Happé, 2019), and (iii) family socio-economic advantage as indexed by higher maternal education and family income as these are associated with a reduced prevalence of ADHD in childhood (Green, McGinnity, Meltzer, Ford, & Goodman, 2005; Hjern, Weitoft, & Lindblad, 2010).

#### Internal resources: verbal and reading ability

Childhood verbal ability was assessed using the Wechsler Intelligence Scale for Children (Wechsler, Golombok, & Rust, 1992) at age 8 years. Reading ability was assessed using the basic reading subtest of the Wechsler Objective Reading Dimensions (Rust, Golombok, & Trickey, 1993) at age 7 years.

#### External resources: family income and maternal education

Family income was measured by mother-report when the child was approximately age 11 years as the average household income including social benefits each week on a 10-point scale from <£120 to ≥£800. Maternal education was assessed by mother-report during pregnancy as the highest educational qualification on a 5-point scale from CSE or no qualifications to university degree.

### Depression risk factors

#### Depression genetic risk

Genetic risk for depression was indexed using PRS based on GWAS of major depression (Wray et al., 2018). Genotyping details as well as full methods for generating the PRS are presented in the Supplementary Material.

#### Maternal depression

History of depression in the mothers was assessed during pregnancy. Assessment was by self-report of a lifetime ever history of severe depression.

### Analyses

Late-onset ADHD was defined using different informants, operationalisations and measures to assess consistency of findings across definitions.

#### Operationalising ADHD onset based on SDQ ADHD symptom cut-points

ADHD symptoms were categorised using a similar procedure to our previous work (Cooper et al., 2018), using Stata 15 (StataCorp, 2017). The SDQ was used for categorical operationalisations as it was administered at more ages than the DAWBA and because there is no recommended cut-point for DAWBA symptoms (the ALSPAC population-based cohort includes too few individuals who met criteria for DSM diagnosis of ADHD to analyse). Separate ADHD groups were generated using (a) self-report adult data, and (b) parent-report adult data based on the recommended cut-points at ages 7, 8, 9, 12, 17 and 25 years (see above). Individuals were categorised as having child-onset ADHD if they had high symptoms at ages 7, 8, 9 or 12 years. Participants identified as having child-onset ADHD were classified as having *child-onset persistent ADHD* if they had high symptoms at either age 17 or 25 years, otherwise they were classified as having *child-limited ADHD.* Individuals with low ADHD symptoms at ages 7, 8, 9 and 12 years, but who had elevated symptom levels at ages 17 or 25 years were classified as having *late-onset ADHD.* Those with subthreshold symptoms in childhood, but high symptoms at ages 17 or 25 were categorised as *subthreshold late-onset* and are presented for descriptive purposes only. Individuals without high symptoms at any time-point were classified as having *low symptoms.* The definitions of all groups are shown in Supplementary Table 1. Analyses were conducted using multiple imputation with inverse probability weighting (IPW/MI)(Seaman, White, Copas, & Li, 2012), including individuals (N=4224) with SDQ-ADHD data available in childhood, adolescence and adulthood (see Supplementary Material for details). Sensitivity analyses based on complete-case analyses and also using IPW (without MI) are shown in the Supplementary Material. Given recent findings (Sibley et al., 2021), post-hoc analyses also examined whether ADHD symptom levels fluctuated in some individuals.

#### Operationalising ADHD onset using a latent trajectory approach

Growth mixture modelling (GMM) was used to derive trajectories of ADHD symptoms from ages 4 to 25 years in Mplus (Muthén & Muthén, 1998-2012) separately using continuous scores for (a) parent-rated SDQ, and (b) parent-rated DAWBA. GMM aims to group individuals into categories based on patterns of change over time (Muthen & Muthen, 2000). Starting with a single k-class solution, k+1 solutions were fitted until the optimum solution was reached. Given the large gap between the adolescent and adult assessments, models were fit for a piecewise growth model with a single intercept and two linear slope factors: one for measurements from ages 4 to 17 years (7 to 15 years for the DAWBA) and one for ages 15/17 (DAWBA/SDQ respectively) and 25 years: the second slope variance was fixed to zero to avoid nonidentification as only two time-points were included in this growth factor. Analyses were run using full information maximum likelihood (FIML), including individuals in the analyses where at least two time-points of data were available: N=9764 for the SDQ and N=8132 for the DAWBA. Sensitivity analyses deriving trajectories based on a range of missing data requirements are shown in the Supplementary Material. Each model was run with 5000 random starting values and 500 optimizations (Muthén & Muthén, 1998-2012). Models were run using a robust maximum likelihood parameter estimator (Muthén & Muthén, 1998-2012) and class sizes are reported based on the estimated model with Ns rounded to the nearest integer.

#### Associations with other variables

We report the means or proportions for each examined variable for each ADHD group. Our primary analyses compared late-onset versus child-onset persistent ADHD. We used multinomial logistic regression to test associations. We used child-onset persistent ADHD as the reference group for the primary analyses and ran secondary analyses using low ADHD symptoms as the reference. For trajectory analyses we used a bias-free three step approach which accounts for measurement error in class assignment (DCAT for estimating proportions, DU3STEP for means and R3STEP for multinomial regression)(Asparouhov & Muthén, 2013). Ordinal family resources variables (family income, maternal education) were entered in regressions as continuous variables. Regressions examining computerised tests of adult ADHD cognitive impairment included device type used (desktop, tablet, mobile) as a covariate.

## Results

### ADHD assessed prospectively defined using different informants, operationalisations and measures

Estimates of late-onset ADHD across definitions are shown in Table 1. Data on comorbid autism, anxiety and depression symptoms by ADHD group are described in the Supplementary Material.

#### SDQ ADHD symptom cut-points

**Figure 1:**
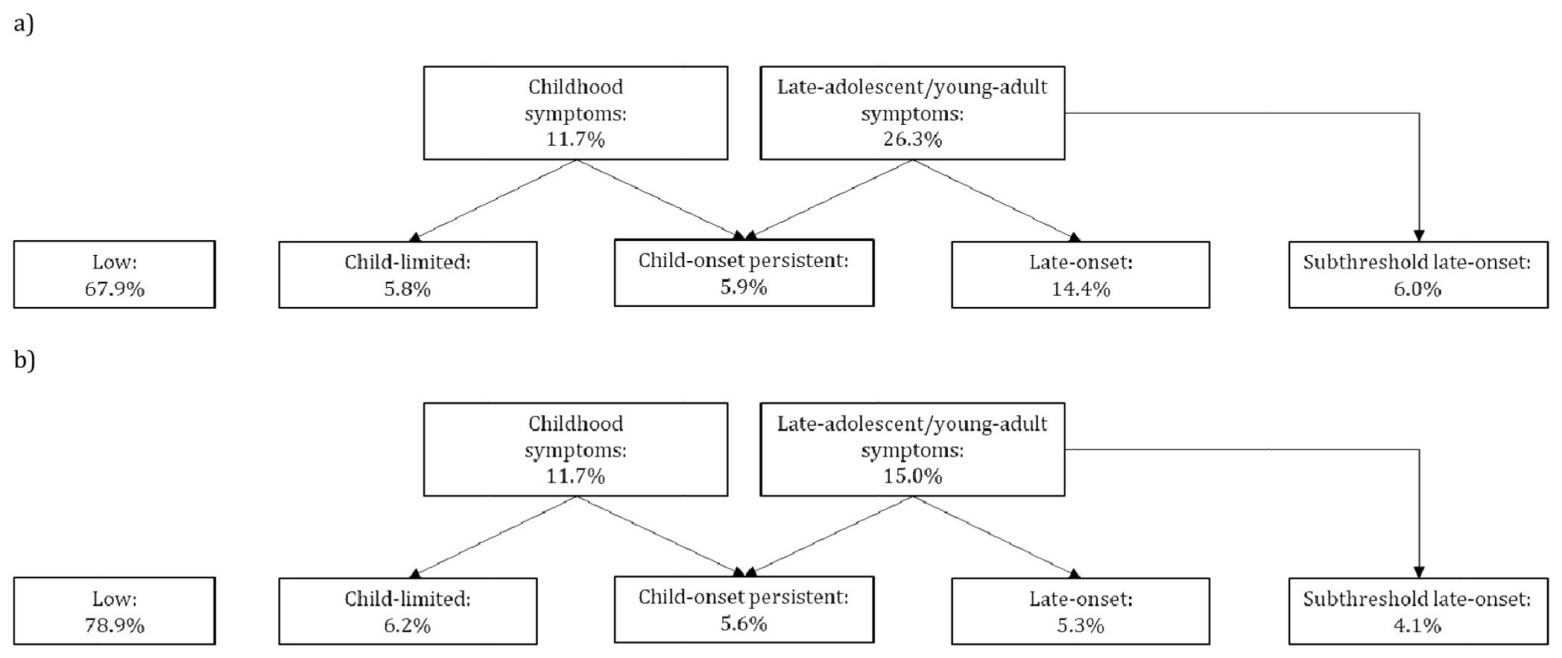
ADHD group based on Strengths and Difficulties Questionnaire cut-points a) Parent-report in childhood and self-report in adulthood b) Parent-report in childhood and adulthood

ADHD groupings based on self- and parent-reported SDQ cut-points in adulthood are shown in Figure 1a and 1b respectively. The vast majority of those with late-onset ADHD had adult onset (96.0% for self-report and 89.3% for parent-report) rather than adolescent onset. ADHD medication use was reported by 3.4% and 5.0% of those in the self-rated and parent-rated late-onset groups respectively, compared to 7.9% and 8.2% of those with child-onset persistent symptoms.

Comparing parent- and self-rated late-onset definitions, 44.6% of those with late-onset ADHD according to parent-reports were also categorised as late-onset based on self-report. In contrast, only 16.6% of those with late-onset ADHD based upon self-report also had late-onset

ADHD by parent-reports. Additional details of overlap between self- and parent-reported adult symptoms are given in the Supplementary Material.

Post-hoc analyses suggested 3.4-6.6% of the sample experienced fluctuating (temporarily-remitted) symptoms; results are presented in the Supplementary Material.

#### A latent trajectory approach: parent-report SDQ and DAWBA

**Figure 2:**
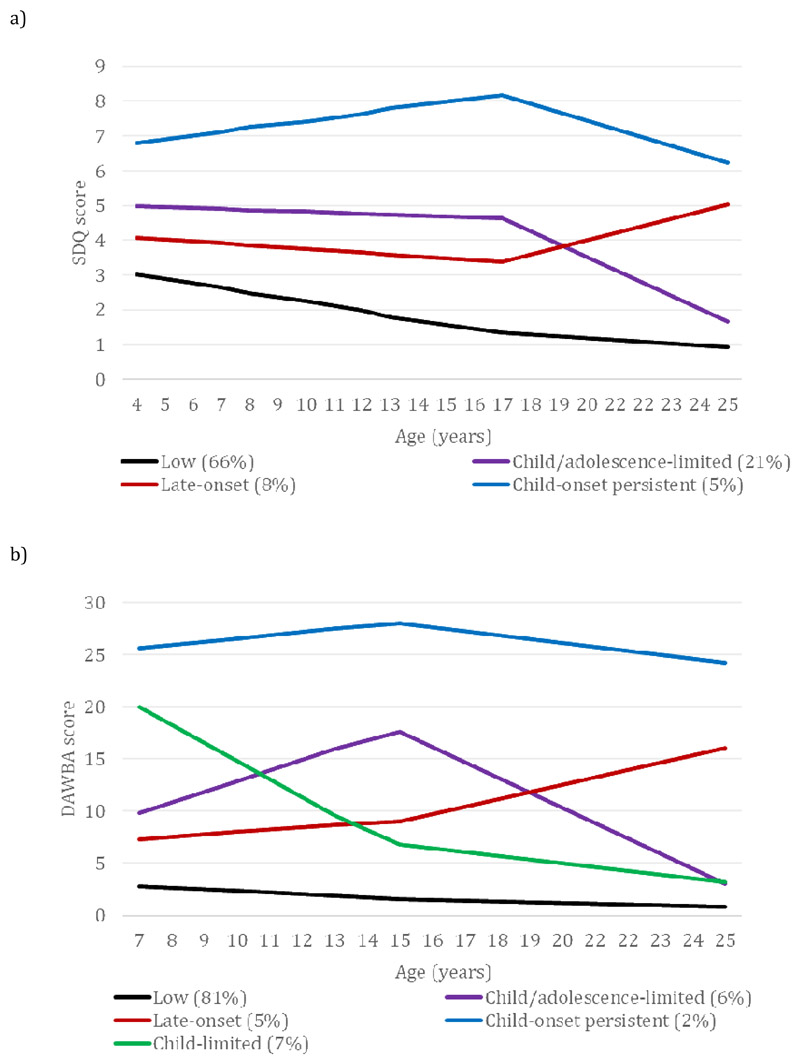
ADHD symptoms by class a) Strengths and Difficulties Questionnaire b) Development and Well-Being Assessment

For trajectory analyses, only parent-reports were used to enable a consistent informant across time points. As shown in Figure 2, we identified four trajectory classes using the parent-rated SDQ and five using the parent-rated DAWBA (see Supplementary Material). ADHD medication use was reported by 2.9% and 3.6% of those in the SDQ and DAWBA late-onset groups respectively, compared to 15.4% and 33.6% of those with child-onset persistent symptoms.

### Testing hypotheses investigating the nature of late-onset ADHD

The means and proportions for examined variables by ADHD group are shown in Table 2. Multinomial odds ratios (relative risk ratios) comparing late-onset to child-onset persistent ADHD are presented in Table 3. Secondary analyses comparing these ADHD groups to those with low ADHD symptoms are shown in Supplementary Tables 2–5.

#### Hypothesis 1: late-onset ADHD reflects a similar phenotype to child-onset ADHD

##### ADHD risk factors

Regardless of informant or classification system, male sex was consistently associated with a decreased likelihood of having late-onset compared to child-onset persistent ADHD. ADHD PRS were similar in those with late-onset and child-onset persistent ADHD when using parent-reported symptoms but were lower in those with late-onset ADHD when using self-reported adult symptoms. We did not find strong evidence that prevalence of preterm birth or low birth weight differed in the late-onset compared to child-onset persistent group.

##### Cognitive tasks

ADHD cognitive task performance did not consistently differ between late-onset and child-onset persistent ADHD when using parent-reported symptoms but was higher in those with late-onset ADHD when using self-reported adult symptoms.

##### Childhood resources

Higher levels of childhood verbal and reading ability, family income and maternal education were associated with late-onset compared to child-onset persistent ADHD.

#### Hypothesis 2: late-onset ADHD reflects a variant of depression

##### Depression risk factors

There was not strong evidence for an association with late-onset ADHD compared to child-onset persistent ADHD for depression PRS or maternal depression. There was also not a female preponderance in the late-onset group.

## Discussion

This study aimed to investigate the nature of late-onset ADHD using different informants, operationalisations and measures in a prospective, longitudinal population sample spanning childhood to young-adulthood. We tested two hypotheses on the nature of late-onset ADHD symptoms: (a) late-onset symptoms reflect a similar phenotype to child-onset ADHD, but with delayed symptom onset, and (b) late-onset ADHD symptoms reflect a variant of depression.

Regardless of ADHD informant, operationalisation or measure, we found evidence for a group of individuals with high levels of ADHD symptoms that start for the first time after childhood. In late-adolescence/young-adulthood, the prevalence of late-onset ADHD was higher for self-reports than for parent-reports. The majority of late-onset ADHD identified was adult-rather than adolescent-onset: while for categorical definitions this could have been due to the lower cut-point used in young-adulthood compared to adolescence, this would not apply to trajectory analyses which used continuous symptom scores. Adult-onset being more common than adolescent-onset may explain why previous trajectory analyses in this same cohort with follow-up to age 17 years did not identify a late-onset trajectory (Riglin et al., 2016) whereas analyses in another sample followed up to age 22 years did (Breda et al., 2021).

Our analyses investigating associations with ADHD and depression risk factors, and internal and external resources generally supported the hypothesis that late-onset ADHD reflects a similar phenotype to child-onset ADHD, rather than a variant of depression when parent-ratings were used. However, findings for self-rated ADHD were not clear. We did not find evidence of a female preponderance in the late-onset group, which would have been expected for depression (Thapar et al., 2012), although those with late-onset ADHD were more likely to be female than those with child-onset persistent ADHD, which is consistent with previous work (e.g. Breda et al., 2021). Regardless of late-onset definition, we did not find evidence for associations with depression PRS (consistent with previous work: Agnew-Blais et al., 2021) and history of maternal depression was similar in the late-onset and child-onset persistent group.

Our “alternative disorder” hypothesis focusses specifically on depression, because this typically onsets at the same age as late-onset ADHD (adolescence or early adulthood) (Thapar et al., 2012) and depression and ADHD symptom overlap means that questionnaire measures of ADHD might detect depression symptomatology. However, there are other possible candidates. For example, although anxiety typically onsets earlier, in childhood (Beesdo, Knappe, & Pine, 2009), it also shares some symptoms with ADHD (e.g. restlessness). Substance use also typically onsets in adolescence or early adulthood (Solmi et al., 2021) and can share some features with ADHD (e.g. concentration problems) although conversely could also mask ADHD symptoms (Katzman, Bilkey, Chokka, Fallu, & Klassen, 2017). Future research may benefit from investigating the relationship between late-onset ADHD and other psychiatric disorders as well as other physical/somatic disorders associated with ADHD such as sleep-disordered breathing (Sedky, Bennett, & Carvalho, 2014).

We found similarly elevated levels of genetic risk for ADHD (indexed by PRS) in those with late-onset ADHD as those with child-onset persistent ADHD when defined using parent-reports, but found lower PRS in those with late-onset ADHD when using self-reported adult symptoms. This PRS finding for parent-reported late-onset ADHD was consistent across different operationalisations and measures of ADHD. Previous studies have not found evidence of elevated ADHD PRS in those with late-onset ADHD: these have either used self-reports (Agnew-Blais et al., 2021; Moffitt et al., 2015) or have followed up to adolescence (age 17 years) rather than young-adulthood (Manfro et al., 2019; Riglin et al., 2016). We also found similar levels of cognitive difficulties (impaired sustained attention and response inhibition) in those with late-onset ADHD as those with child-onset persistent ADHD when defined using parent-reports but found higher cognitive performance in those with late-onset ADHD when using self-reported adult symptoms. Given the relatively limited overlap between self- and parent-reported ADHD symptoms (Barkley et al., 2002) and the higher proportion of individuals identified in this sample as having high ADHD symptoms in young-adulthood using self-reports compared to parent-reports (Riglin, Agha et al. Riglin et al., 2021; 2020), late-onset cases identified by self-report likely captures a broader group, which may include those whose symptoms are more scaffolded/compensated for and therefore less well observed by others. Additionally, ADHD PRS were derived from a genome-wide association study of childhood ADHD where diagnoses will typically have been made using parent-reports so may not capture genetic variants associated with self-reported ADHD symptoms. Across late-onset definitions, we did not find strong evidence that the ADHD risk factors of preterm birth and low birth weight differed between the late-onset ADHD and the child-onset persistent ADHD, although this may reflect limited power for these analyses. Interestingly, ADHD medication use was reported by those with late-onset ADHD (although not to the same extent as those with child-onset persistent ADHD). We cannot conclude from our data whether medication has been effective: a crucial question is whether usual ADHD treatment is effective for those with late-onset ADHD.

One explanation for why the same underlying disorder (ADHD) may onset later in development for some is that higher levels of childhood internal and external resources may allow individuals to compensate for underlying ADHD difficulties or obscure/delay the presence of symptoms (i.e. compensation or scaffolding). This is supported by reported associations between late-onset ADHD and higher IQ (Breda et al., 2021; Kosaka et al., 2019) and is similar to the growing evidence that some individuals with neurodevelopmental conditions such as autism may show later manifestation of symptoms due to earlier compensation or scaffolding (Livingston & Happé, 2017). We investigated this hypothesis by examining possible childhood resources including both individual-based and family-based factors. Our findings suggest children’s verbal ability, reading ability, family income and maternal education are higher in those with late-onset compared to child-onset persistent ADHD. These findings are consistent with the hypothesis that higher individual and family resources may enable individuals to compensate or scaffold childhood ADHD liability, but that symptoms later emerge as demands on independence increase and family resources become more distal. Further research into the possible relationship between individual characteristics, family resources and ADHD symptom age-at-onset is needed. Future studies would benefit from including measures that capture more specific childhood resources to provide insight into possible mechanisms as well as potential targets to help scaffold ADHD difficulties in childhood.

Our findings should be considered in the context of study limitations. Like many population cohorts, ALSPAC suffers from non-random attrition, whereby individuals at elevated risk of psychopathology are more likely to drop-out of the study (Martin et al., 2016; Taylor et al., 2018). Thus, prevalence rates need to be viewed with caution; however associations are more robust to missingness. We used different approaches to missing data including multiple imputation with inverse probability weighting and full information maximum likelihood to try to minimise the effect of missingness, although these assume that missingness is independent of the unobserved missing data (given the variables in the model) and that the imputation model is correctly specified. Our consistent findings across different approaches to missing data (for parent-reported late-onset) add confidence to our findings. The use of a population cohort also means a limited number of individuals in the high-symptom groups, that reduces power to detect smaller effect size associations and association with less prevalent risk factors such as preterm birth. It is also important to note that findings from our multinomial regressions comparing late-onset and child-onset persistent ADHD might suffer from selection bias: conditioning on not having ADHD in adolescence/young-adulthood could induce associations via unmeasured confounding (collider bias) (Cole et al., 2010). If it is known an individual has ADHD in adolescence/young-adulthood, these comparisons are valid for comparing characteristics of late-onset versus child-onset (persistent) ADHD. Finally, our study examined ADHD symptoms rather than DSM-5 diagnoses. In particular the SDQ, whilst a valid screening tool, does not assess the full range of DSM-5 ADHD symptoms. While evidence suggests that ADHD symptoms behave as a continuously distributed dimension with regards to risk factors and with adverse outcomes (Thapar & Cooper, 2016), we cannot rule out that assessments based on clinician assessments (for whom age-at-onset is based on retrospective reports) may find a different pattern of results.

In summary, across different operationalisations and measures, we found similar levels of ADHD genetic liability (PRS) in those with parent-rated late-onset compared to child-onset persistent ADHD, suggesting that parent-rated late-onset ADHD is indexing ADHD genetic liability. Those with late-onset ADHD had higher levels of individual-based and family-based resources. Self-ratings however defined a different late-onset ADHD group which did not show the same profile as child-onset ADHD. Taken together our findings suggest that at least for some, ADHD may onset later in development in the context of childhood compensation or scaffolding resources and further challenges the assertion that ADHD always first onsets before age 12 years.

## Supplementary Material

Supplementary Material

## Figures and Tables

**Table 1:** Prevalence of late-onset and child-onset persistent ADHD using different informants, operationalisations and measures

	Late-onset ADHD	Child-onset persistent ADHD
SDQ cut-points: self-reports#	14.4%	5.9%
SDQ cut-points: parent-reports	5.3%	5.6%
Latent trajectories: SDQ	8.2%	5.3%
Latent trajectories: DAWBA	4.8%	1.9%

SDQ = Strengths and Difficulties Questionnaire, DAWBA = Development and Well-Being Assessment.

# Self-reports in adulthood, parent-reports for prior assessments. Trajectory analyses based on parent-report. Cut-point based definitions using multiple imputation with inverse probability weighting, trajectory definitions using full information maximum likelihood.

**Table 2:** ADHD-risk factors, cognitive tasks, childhood resources and depression risk factors means or proportions by ADHD group

	SDQ cut-points: self-reports^#^					SDQ cut-points: parent-reports					Latent trajectories: SDQ				Latent trajectories: DAWBA				
	Low	CL	COP	LO	SO	Low	CL	COP	LO	SO	Low	CAL	COP	LO	Low	CL	CAL	COP	LO
*ADHD risk factors*																			
Male sex	0.46 (0.01)	0.65 (0.04)	0.71 (0.04)	0.50 (0.03)	0.64 (0.04)	0.47 (0.01)	0.62 (0.04)	0.74 (0.04)	0.48 (0.04)	0.65 (0.05)	0.43 (0.01)	0.66 (0.02)	0.79 (0.02)	0.52 (0.03)	0.46 (0.01)	0.68 (0.02)	0.66 (0.03)	0.76 (0.05)	0.59 (0.03)
ADHD PRS	-0.08 (0.03)	0.10 (0.01)	0.23 (0.01)	-0.08 (0.05)	0.18 (0.10)	-0.09 (0.02)	0.03 (0.09)	0.32 (0.12)	0.14 (0.09)	0.16 (0.12)	-0.11 (0.02)	0.05 (0.04)	0.24 (0.08)	0.17 (0.08)	-0.09 (0.02)	0.14 (0.06)	0.11 (0.07)	0.32 (0.12)	0.21 (0.09)
Preterm birth	0.04 (0.00)	0.12 (0.05)	0.06 (0.03)	0.04 (0.01)	0.06 (0.03)	0.04 (0.00)	0.07 (0.03)	0.11 (0.04)	0.04 (0.02)	0.07 (0.03)	0.04 (0.00)	0.05 (0.01)	0.09 (0.02)	0.07 (0.02)	0.04 (0.00)	0.06 (0.01)	0.06 (0.01)	0.08 (0.02)	0.05 (0.02)
Low birth weight	0.04 (0.00)	0.06 (0.03)	0.03 (0.02)	0.05 (0.01)	0.07 (0.03)	0.04 (0.00)	0.05 (0.03)	0.05 (0.03)	0.03 (0.02)	0.07 (0.03)	0.04 (0.00)	0.05 (0.01)	0.07 (0.01)	0.04 (0.01)	0.04 (0.00)	0.06 (0.01)	0.06 (0.01)	0.07 (0.02)	0.05 (0.02)
*Cognitive tasks*																			
Child sustained attention	0.04 (0.03)	-0.13 (0.10)	-0.21 (0.11)	-0.01 (0.05)	-0.12 (0.11)	0.03 (0.02)	-0.11 (0.09)	-0.23 (0.12)	-0.02 (0.09)	-0.13 (0.15)	0.16 (0.02)	0.00 (0.07)	-0.31 (0.12)	-0.92 (0.10)	0.12 (0.01)	-0.61 (0.14)	0.03 (0.05)	-0.28 (0.12)	-0.80 (0.22)
Child response inhibition	0.04 (0.03)	-0.15 (0.12)	-0.20 (0.11)	0.01 (0.07)	-0.04 (0.09)	0.04 (0.02)	-0.11 (0.10)	-0.25 (0.12)	-0.11 (0.08)	-0.08 (0.11)	0.15 (0.02)	-0.11 (0.08)	-0.20 (0.11)	-0.70 (0.10)	0.08 (0.01)	-0.19 (0.07)	-0.60 (0.12)	-0.35 (0.13)	-0.02 (0.07)
Adult sustained attention	-0.02 (0.05)	-0.25 (0.16)	-0.39 (0.17)	-0.20 (0.08)	-0.31 (0.13)	-0.04 (0.05)	-0.29 (0.14)	-0.35 (0.20)	-0.29 (0.14)	-0.36 (0.18)	0.07 (0.04)	0.07 (0.16)	-0.44 (0.31)	-0.36 (0.28)	0.07 (0.03)	-0.26 (0.22)	-0.54 (0.29)	0.36 (0.41)	-0.03 (0.15)
Adult response inhibition	-0.07 (0.04)	-0.33 (0.15)	-0.54 (0.16)	-0.17 (0.08)	-0.36 (0.14)	-0.08 (0.04)	-0.31 (0.13)	-0.57 (0.19)	-0.35 (0.13)	-0.44 (0.16)	0.11 (0.03)	-0.20 (0.14)	-0.72 (0.41)	-0.28 (0.19)	0.07 (0.03)	-0.20 (0.19)	-0.29 (0.23)	0.41 (0.48)	-0.38 (0.26)
*Childhood resources*																			
Verbal ability	107.97 (0.40)	101.40 (1.57)	99.45 (1.58)	109.09 (0.98)	103.64 (1.69)	108.43 (0.37)	101.69 (1.50)	99.00 (1.63)	104.29 (1.72)	101.31 (1.99)	109.96 (0.28)	102.45 (0.74)	98.47 (1.26)	105.47 (1.32)	109.48 (0.23)	103.33 (1.10)	101.03 (1.14)	96.76 (1.87)	102.28 (1.38)
Reading ability	29.27 (0.23)	24.48 (0.93)	23.04 (1.00)	30.03 (0.51)	26.86 (0.99)	29.48 (0.21)	25.14 (0.86)	22.22 (1.05)	28.20 (0.78)	25.69 (1.22)	30.32 (0.16)	24.72 (0.38)	21.18 (0.68)	27.09 (0.75)	29.70 (0.13)	24.08 (0.60)	24.07 (0.58)	20.04 (1.02)	26.03 (0.76)
Family income	6.78 (0.07)	6.18 (0.26)	5.72 (0.25)	6.84 (0.15)	6.12 (0.26)	6.81 (0.06)	6.38 (0.23)	5.47 (0.27)	6.43 (0.26)	5.95 (0.31)	7.06 (0.05)	6.35 (0.11)	5.96 (0.20)	6.97 (0.17)	7.03 (0.04)	6.47 (0.17)	6.37 (0.17)	5.73 (0.27)	6.33 (0.23)
Maternal education	2.91 (0.03)	2.67 (0.13)	2.55 (0.13)	2.98 (0.07)	2.76 (0.12)	2.93 (0.03)	2.66 (0.11)	2.56 (0.13)	2.84 (0.11)	2.71 (0.14)	3.19 (0.02)	2.91 (0.04)	2.71 (0.08)	3.73 (0.06)	3.27 (0.02)	3.21 (0.07)	3.02 (0.07)	2.87 (0.11)	3.23 (0.10)
*Depression risk factors*																			
Depression PRS	-0.05 (0.03)	-0.02 (0.11)	0.14 (0.11)	0.03 (0.05)	0.08 (0.10)	-0.03 (0.02)	0.08 (0.10)	0.04 (0.12)	0.04 (0.08)	-0.10 (0.12)	-0.04 (0.02)	-0.02 (0.04)	0.03 (0.07)	0.08 (0.07)	-0.04 (0.02)	0.07 (0.06)	-0.04 (0.06)	0.05 (0.11)	-0.03 (0.08)
Maternal depression	0.08 (0.01)	0.09 (0.04)	0.14 (0.04)	0.11 (0.02)	0.13 (0.04)	0.07 (0.01)	0.09 (0.03)	0.14 (0.05)	0.20 (0.04)	0.20 (0.05)	0.06 (0.00)	0.11 (0.01)	0.17 (0.02)	0.13 (0.02)	0.06 (0.00)	0.12 (0.02)	0.11 (0.02)	0.15 (0.03)	0.18 (0.03)

SDQ = Strengths and Difficulties Questionnaire, DAWBA = Development and Well-Being Assessment. CL=child-limited, CAL=child/adolescent-limited, COP=child-onset persistent, LO=late-onset, SO=subthreshold late-onset. Standard errors in parentheses. Cut-point based analyses using multiple imputation with inverse probability weighting, trajectory definitions using full information maximum likelihood to derive trajectories and listwise deletion for associations with other variables.

# Self-reports in adulthood, parent-reports for prior assessments. Adult cognitive tasks not controlling for device type. SDQ trajectory means for adult response inhibition and family income estimated using BCH instead of DU3STEP to avoid class formation changes (Asparouhov & Muthén, 2014). Means presented for PRS, cognitive tasks and childhood resources (family income assessed on a 1-10 scale and maternal education on a 1-5 scale), proportions presented for male sex, preterm birth, low birth weight and maternal depression.

**Table 3:** Comparing late-onset to child-onset persistent ADHD: associations for ADHD-risk factors, cognitive tasks, childhood resources and depression risk factors

	SDQ cut-points: self-reports^#^ OR (95% CI)	SDQ cut-points: parent-reports OR (95% CI)	Latent trajectories: SDQ OR (95% CI)	Latent trajectories: DAWBA OR (95% CI)
*ADHD risk factors*				
Male sex	0.41 (0.29-0.58), p<0.001	0.31 (0.20-0.47), p<0.001	0.22 (0.15-0.33), p<0.001	0.45 (0.28-0.73), p=0.001
ADHD PRS	0.70 (0.58-0.85), p=0.01	0.83 (0.66-1.04), p=0.11	0.91 (0.73-1.14), p=0.43	0.89 (0.67-1.20), p=0.46
Preterm birth	0.51 (0.23-1.14), p=0.10	0.54 (0.21-1.35), p=0.19	0.83 (0.43-1.60), p=0.58	0.44 (0.16-1.25), p=0.13
Low birth weight	1.04 (0.43-2.49), p=0.94	0.38 (0.11-1.28), p=0.12	0.81 (0.44-1.50), p=0.51	0.55 (0.20-1.51), p=0.25
*Cognitive tasks*				
Child sustained attention	1.23 (1.04-1.46), p=0.02	1.25 (1.02-1.54), p=0.03	1.48 (1.06-2.06), p=0.02	1.04 (0.82-1.32), p=0.74
Child response inhibition	1.22 (1.03-1.45), p=0.02	1.10 (0.91-1.33), p=0.34	1.29 (0.97-1.71), p=0.08	1.20 (0.95-1.53), p=0.13
Adult sustained attention	1.26 (0.99-1.61), p=0.06	1.13 (0.84-1.50), p=0.42	1.18 (0.49-2.87), p=0.71	0.58 (0.22-1.50), p=0.26
Adult response inhibition	1.34 (1.08-1.69), p=0.01	1.22 (0.94-1.59), p=0.14	1.48 (0.67-3.27), p=0.30	0.40 (0.05-3.37), p=0.40
*Childhood resources*				
Verbal ability	1.03 (1.02-1.04), p<0.001	1.02 (1.01-1.04), p=0.001	1.03 (1.01-1.04), p<0.001	1.02 (1.00-1.04), p=0.01
Reading ability	1.08 (1.06-1.10), p<0.001	1.07 (1.05-1.10), p<0.001	1.05 (1.01-1.08), p=0.01	0.99 (0.96-1.01), p=0.37
Family income	1.14 (1.06-1.22), p<0.001	1.11 (1.02-1.21), p=0.02	1.15 (1.07-1.25), p<0.001	1.10 (1.00-1.21), p=0.06
Maternal education	1.27 (1.11-1.46), p=0.001	1.11 (0.94-1.32), p=0.22	1.50 (1.31-1.72), p<0.001	1.25 (1.04-1.49), p=0.02
*Depression risk factors*				
Depression PRS	0.90 (0.74-1.09), p=0.30	0.98 (0.77-1.23), p=0.84	1.03 (0.85-1.25), p=0.76	0.92 (0.71-1.20), p=0.53
Maternal depression	0.91 (0.47-1.79), p=0.79	1.65 (0.79-3.44), p=0.18	0.58 (0.34-1.00), p=0.05	1.26 (0.67-2.36), p=0.47

SDQ = Strengths and Difficulties Questionnaire, DAWBA = Development and Well-Being Assessment. 95% confidence intervals in parentheses. Multinomial odds ratios with child-onset persistent ADHD as the reference, based on ADHD groups as the outcome regardless of temporal precedence. Cut-point based analyses using multiple imputation with inverse probability weighting, trajectory definitions using full information maximum likelihood to derive trajectories and listwise deletion for associations with other variables.

# Self-reports in adulthood, parent-reports for prior assessments.
