# Supplementary material for: “Late-onset” ADHD symptoms in young adulthood: is this ADHD?
Source: J Atten Disord. Author manuscript; Available in PMC 2022 Aug 1. (PMC7612898; doi:10.1177/10870547211066486)
Supplement: Supplementary Material [file EMS140021-supplement-Supplementary_Material.docx]

**Supplementary Material**

**The Avon Longitudinal Study of Parents and Children (ALSPAC)**

Pregnant women resident in Avon, UK with expected dates of delivery 1st April 1991 to 31st December 1992 were invited to take part in the study. The initial number of pregnancies enrolled is 14,541 (for these at least one questionnaire has been returned or a “Children in Focus” clinic had been attended by 19/07/99). Of these initial pregnancies, there was a total of 14,676 foetuses, resulting in 14,062 live births and 13,988 children who were alive at 1 year of age. When the oldest children were approximately 7 years of age, an attempt was made to bolster the initial sample with eligible cases who had failed to join the study originally. As a result, the total sample size for data collected after the age of seven is therefore 15,454 pregnancies, resulting in 15,589 foetuses. Of these 14,901 were alive at 1 year of age. Part of this data was collected using REDCap (Harris et al., 2019; Harris et al., 2009). Ethical approval for the study was obtained from the ALSPAC Law and Ethics Committee and Local Research Ethics Committees. Informed consent for the use of data collected via questionnaires and clinics was obtained from participants following the recommendations of the ALSPAC Ethics and Law Committee at the time. Consent for biological samples has been collected in accordance with the Human Tissue Act (2004). Please note that the study website contains details of all the data that is available through a fully searchable data dictionary and variable search tool: http://www.bristol.ac.uk/alspac/researchers/our-data/. Further details of the study, measures and sample can be found elsewhere (Boyd et al., 2013; Fraser et al., 2013; Northstone et al., 2019). Where families included multiple births, we included the oldest sibling.

**Generating polygenic scores**

Polygenic scores (PRS) were derived for 9,912 ALSPAC children who were genotyped using the Illumina HumanHap500-quad genotyping array. Individuals were excluded based on gender mismatches, minimal or excessive heterozygosity, genotype missingness (>3%), insufficient sample replication (IBD <0.8), non-European ancestry (assessed by multidimensional scaling analysis and compared with Hapmap II) and cryptic relatedness (IBD > 0.1). SNPs were excluded based on minor allele frequency (<1%), call rate (<95%) or evidence for violations of Hardy-Weinberg equilibrium (P < 5E-7). Imputation was conducted by the ALSPAC team using Impute V2.2.2 against the 1000 genomes reference panel (Phase 1, Version 3: all polymorphic SNPs excluding singletons), using all 2,186 reference haplotypes (including non-Europeans). Best guess SNPs were subsequently filtered based on minor allele frequency (<1%) and imputation quality (INFO<0.8).

Genome-wide association study (GWAS) were filtered to remove SNPs that were palindromic, insertions/deletions, non-autosomal, INFO score <0.8, missing in N>1 study and duplicates (https://github.com/ricanney). Depression results for 23andme (75,607 cases and 231,747 controls)(Hyde et al., 2016) and the other samples included in the latest depression GWAS(Wray et al., 2018) (PGC29, deCODE, Generation Scotland, GERA, iPSYCH, and UK Biobank) were meta-analysed in METAL.

PRS were generated for individuals in ALSPAC as the number of disorder risk alleles – defined using the GWAS summary statistics - weighted by effect size, using PRSice version 1.25 (Euesden, Lewis, & O'Reilly, 2015); SNPs were clumped with an R^2^ threshold of 0.1 and a distance threshold of 1000kb and excluding the extended major histocompatibility complex (MHC; chromosome 6: 26-33Mb) due to the high linkage disequilibrium (LD) within this region. PRS were generated using GWAS of ADHD (19,099 cases and 34,194 controls)(Demontis et al., 2019) and depression (135,458 cases and 344,901 controls)(Wray et al., 2018) with risk alleles defined as those associated with case-status at p<0.05. ALSPAC was not included in either GWAS. Polygenic risk scores were standardized to mean=0 SD=1.

**Cognitive tasks**

In childhood cognitive tasks were completed as part of a ‘Focus’ clinic at approximately age 8.5 years. In young-adulthood, participants who completed the ALSPAC questionnaire at age 25 years were invited to also complete cognitive tasks online so these data are available for a subsample of those with age 25 self-report questionnaire data (see Supplementary Table 6).

Sustained attention was assessed in childhood using the Tests of Everyday Attention for Children (TEA-Ch)(Robertson, Ward, Ridgeway, & Nimmo-Smith, 1996) Sky Search task, in which the participant is required to identify identical pairs of spaceships. Scores are derived by subtracting the mean time for the control condition (motor score: no non-identical pairs) from the experimental condition (including non-identical pairs). Young-adult sustained attention was assessed using the Sustained Attention Task (SART)(Bellgrove, Hawi, Kirley, Gill, & Robertson, 2005). The SART is a go/no-go computer-based assessment of working memory, sustained attention and impulse control. Participants are instructed to respond to the presentation of some digits (go-trials) whilst refraining from responding when others were presented (no-go trial). Child and young-adult sustained attention scores were transformed using log 10 due to skew, multiplied by minus one so that higher scores reflect better cognitive performance and subsequently standardized to mean=0 SD=1 to aid interpretation.

Inhibitory control in childhood was assessed using the TEA-Ch Opposite Worlds task, which is a type of Stroop task (Stroop, 1935). Children were instructed to give a verbal response to a specified digit when it appeared on screen, with varying congruency between the digit and the verbal response. Scores are derived by subtracting the mean time for the control condition (Same Worlds trials) from the experimental condition (Opposite Worlds trials). Response inhibition in adulthood was assessed using the Double Trouble task (Metzler-Baddeley, Caeyenberghs, Foley, & Jones, 2016), also a Stroop-based task. Participants are instructed to select one of two words that correctly describes the colour of a third word, with varying congruency between font colour and colour meaning. Child response inhibition scores were multiplied by minus one so that both child and young-adult higher scores reflect better cognitive performance; scores were subsequently standardized to mean=0 SD=1 to aid interpretation.

**Multiple imputation with inverse probability weighting**

Individuals with ADHD SDQ data in childhood (age 7, 8, 9 or 12 years), late adolescence (age 17 years) and adult (self- or parent-report at age 25 years) data were included in our cut-point bases analyses (N=4,224). Primary ADHD symptoms data (those used to generate age-at-onset groups) were available for 70%-100% of the sample, with additional measures (ADHD medication, ADHD risk factors, cognitive tasks, childhood resources, depression risk factors, other psychopathologies) available for 66-96% with the exceptions of adult cognitive impairment data which was available for 23-25% of the sample. Everyone in the primary sample had at least partial ADHD data (44% complete) and partial covariate data (7% complete). There was a general trend for higher ADHD symptoms and risk factors and lower cognitive tasks and childhood resources to be associated with missing data (Supplementary Table 6).

Missing data were addressed by combining multiple imputation with inverse probability weighting (IPW/MI)(Seaman, White, Copas, & Li, 2012), weighted to the “full” ALSPAC sample (N=14,686, i.e. including those without ADHD data in childhood, adolescence and adulthood). First, inverse probability weighting (IPW)(Seaman & White, 2013) was used to generate weights derived from a logistic regression analysis of missing ADHD data for a set of measures assessed in or soon after pregnancy that were that showed independent association with missing data (see Supplementary Table 7). Minimal missing data on indicators used to derive weights were singly imputed as the modal or mean value (all indicators had <20% of values missing). Weights were stabilized whereby the numerator was the probability of inclusions in the sample (i.e. 4244/14686)(Sayon-Orea et al., 2020). The Hosmer-Lemeshow test was used assess the fit of the missingness model; results did not indicate poor fit (Hosmer-Lemeshow χ2(8)=10.04, p=0.26). For the included sample (N=4,224) weights ranged from 0.36 to 8.04. Second, for those with sufficient ADHD data (i.e. N=4,224) multiple imputation by chained equations (White, Royston, & Wood, 2011) was used to impute missing data for ADHD, comorbidity, ADHD risk factors, cognitive tasks, childhood resources and depression risk factors. The IPW weight, variables that predicted ADHD data missingness (Supplementary Table 7), variables in the analysis models (Supplementary Table 6) and additional measures associated with mental health and impairment (parent-rated social-communication problems at ages 10-25 years, parent-rated ADHD, emotional and conduct problems ages 4-25 years and self-rated at 25 years, self-rated depressive symptoms at ages 10-23 years, sustained attention and response inhibition at age 11 years and school attainment indexed by GCSE points) were included in an imputation model. 250 imputed datasets were generated, which was estimated to be a sufficient number of imputations to ensure that standard errors would not change considerably if the data were imputed again (the recommended 2-stage quadratic rule based on the initial imputation of 250 datasets suggested 9-15 imputations were needed to estimate the ADHD groups and age-at onset, 8-134 for estimated proportions/means of associated variables and 8-111 for multinomial associations with child-onset persistent ADHD as the reference group (von Hippel, 2018). Estimates were combined across imputed datasets using Rubin’s rules (White et al., 2011) using a robust estimator due to uncertainty in weights (Seaman et al., 2012).

**Variable patterns of remission**

Given recent findings (Sibley et al., 2021), post-hoc analyses tested for fluctuating ADHD symptom levels. These analyses used the SDQ ADHD symptom cut-points operationalisation of ADHD onset. At each assessment (ages 7, 8, 9, 12, 17 and 25 years), individuals were either categorised as having *low symptoms* (low or subthreshold symptoms; symptoms also not high at prior assessments), *first-onset* (high symptoms; for the first time at assessment), *persistent* (high symptoms; not for the first time at assessment), *remitted* (low or subthreshold symptoms; symptoms had been high at a previous assessment and are not high at future assessment), *subthreshold* (subthreshold symptoms; symptoms had been high at a previous assessment and are high at future assessment) or *temporarily-remitted* (low symptoms; symptoms had been high at a previous assessment and are high at future assessment).

The proportion of individuals in each of these categories at each assessment are shown in Supplementary Figure 1: across ages 8 to 17 years, 0.3%-2.6% of the sample were classified as *temporarily-remitted* (i.e. had low symptoms but showed high symptoms at prior and subsequent assessments). Combining these assessments suggests the total of sample who had temporarily-remitted (“fluctuating”) symptoms was 3.4% for self-reported adult symptoms and 6.6% for parent-reported symptoms.

**Autism, anxiety and depression symptoms**

Autism symptoms were measured using the parent-rated Social Communication Disorders Checklist (SCDC; cut-point of ≥9)(Skuse, Mandy, & Scourfield, 2005) at age 7 years and parent- and self-rated 28-item version of the Autism-Spectrum Quotient (AQ-28) at age 25 years (“stringent” cut-point ≥70 (Hoekstra et al., 2011)). Anxiety symptoms were measured using the parent-rated emotional problems subscale of the SDQ (cut-point ≥5)(Goodman, 1997) at age 12 years and the self-report Screen for Adult Anxiety Related Disorders (SCAARED; cut-point ≥23)(Angulo et al., 2017) at age 25 years. Depression symptoms were assessed by self-report at ages 12 and 25 years using the short Moods and Feelings Questionnaire (MFQ; cut-point ≥12)(Angold et al., 1995; Thabrew, Stasiak, Bavin, Frampton, & Merry, 2018).

Autism, anxiety and depression symptoms by ADHD group are shown in Supplementary Figure 2. Autism, anxiety and depression were all generally elevated in the late-onset groups compared to those with low ADHD symptoms. Compared to those with child-onset persistent ADHD symptoms, those with late-onset symptoms tended to have lower levels of comorbid autism symptoms but similar levels of depression and adult anxiety.

**Overlap between self- and parent-reported symptoms in young-adulthood**

Based on the SDQ ADHD subscale (Goodman, 1997) an estimated 24.8% of the sample had high self-reported ADHD symptoms in young-adulthood. Of those with high self-rated symptoms, 27.3% also had high parent-reported ADHD symptoms in young-adulthood: this was 13.6% in those with self-rated late-onset ADHD and 60.1% in those with self-rated child-onset persistent ADHD. Conversely, an estimated 13.9% of the sample had high parent-reported ADHD symptoms in young-adulthood, of whom 48.6% also had high self-reported ADHD symptoms in young-adulthood: this was 37.7% in those with parent-rated late-onset ADHD and 56.0% in those with parent-rated child-onset persistent ADHD.

**Selecting the number of trajectories**

Growth mixture modelling was used to derive developmental trajectories of ADHD symptoms spanning ages 4-25 years in Mplus (Muthén & Muthén, 1998-2012) using (a) the parent-rated SDQ (Goodman, 1997), and (b) the parent-rated DAWBA (Goodman, Ford, Richards, Gatward, & Meltzer, 2000). To select the number of classes for the two growth mixture models (GMMs), we initially modelled a single k-class solution, modelling subsequent k+1 solutions until the optimum solution was reached. Each model was run with 5000 random starting values and 500 optimizations (STARTS = 5000 500 in Mplus) (Muthén & Muthén, 1998-2012). Models were fit for a piecewise growth model with a single intercept and two linear slope factors: one for measurement spanning ages 4 to 17years (7 to 15 years for the DAWBA) and one for ages 15/17 (DAWBA/SDQ respectively) and 25 years: the second slope variance was fixed to zero to avoid nonidentification as only two time-points were included in this growth factor.

For the SDQ, model fit significantly improved, as indicated by the fall in loglikelihood value, sample size adjusted Bayesian information criterion and Bootstrapped Likelihood Ratio Test, from the one- to five-class solutions (see Supplementary Table 8). The Vuong-Lo-Mendell-Rubin Likelihood Ratio Rest indicated no significant improvement in model fit from the two- to three-class solution, however given the theoretical justification for identifying more than two classes, we continued modelling additional classes. Subsequently the Vuong-Lo-Mendell-Rubin Likelihood Ratio Rest indicated no significant improvement in model fit from the four- to five-class solution: the four-class solution was therefore selected.

For the DAWBA, model fit significantly improved, as indicated by the fall in loglikelihood value, sample size adjusted Bayesian information criterion, Vuong-Lo-Mendell-Rubin Likelihood Ratio Rest and Bootstrapped Likelihood Ratio Test, from the one- to six-class solutions (also shown in 3). The Vuong-Lo-Mendell-Rubin Likelihood Ratio Rest indicated no significant improvement in model fit from the five- to six-class solution: the five-class solution was therefore selected.

**Missing data sensitivity analyses**

*SDQ ADHD symptom cut-points using complete-case analyses and inverse probability weighting*

Primary analyses operationalising ADHD onset based on SDQ ADHD symptom cut-points were conducted using multiple imputation with inverse probability weighting (IPW/MI)(Seaman et al., 2012), including individuals with any SDQ-ADHD data available in childhood, adolescence and adulthood (see Supplementary Material for details): N=4224, weighted to the “full” ALSPAC sample. We compared the proportion of individuals in the different ADHD groupings using complete case analysis (N=1873: those with parent-reported SDQ at ages 7, 8, 9, 12, 17 and 25 and self-reported SDQ at age 25 years) and using inverse probability weighting (IPW) to “weight” those with complete cases to those included in our primary sample (N=4224) (Seaman & White, 2013). IPW was used to generate weights derived from a logistic regression analysis of missing ADHD data for a set of measures with minimal missingness that showed association with missing data (child sex, maternal depression, age and education, family income). The Hosmer-Lemeshow test did not indicate poor fit (Hosmer-Lemeshow χ2(10)=14.83, p=0.06). For the included sample (N=1,873) weights ranged from 1.50 to 5.13. Estimated proportions across these approaches are shown in Supplementary Table 9: a lower proportion of individuals were estimated to have high ADHD symptoms when using complete cases, with intermediate levels when using IPW compared to the primary analyses using IPW/MI. The lower proportion of the sample estimated to have childhood symptoms when using complete cases meant that of those with late-adolescent/young-adult symptoms, a higher proportion were categorised as late-onset for complete case and IPW estimates.

*Deriving trajectories based on varying levels of missingness*

Sensitivity analyses were also conducted deriving trajectories with varying levels of missingness. Models were run using full information maximum likelihood estimation (FIML) which assumes that data are missing at random (or missing completely at random) conditional on the variable in the model. Primary analyses included individuals in the analyses where at least two time-points of data were available: we re-ran sensitivity analyses requiring (a) requiring only one time-point, and (b) requiring complete data. Models with more stringent inclusion criteria are likely to be at increased risk of bias, arising from increasing differences between missing and non-missing values. For the SDQ, fit statistics showed a similar pattern across varying levels of missingness (Supplementary Table 10) and the selected 4-class solution was similar across these (Supplementary Figure 3). For the DAWBA, using the less stringent missing data criteria (requiring only one time-point), model fit significantly improved as indicated by the fall in loglikelihood value, sample size adjusted Bayesian information criterion and Bootstrapped Likelihood Ratio Test from the one- to six- class solutions, but the Vuong-Lo-Mendell-Rubin Likelihood Ratio Rest indicated little improvement in model fit following the four-class solution (Supplementary Table 11). Comparison of the four- and five-class solutions (shown in Supplementary Figure 4a and 3b) suggested the five-class solution may be more theoretically meaningful because, (i) the four-class solution included an ‘intermediate’ class which showed an adolescent ’dip’ in symptoms instead of the child-limited and late-onset classes identified in the five-class solution, and (ii) that the five-class solution captured groups with higher DAWBA scores. This five-class solution was very similar to that used in the primary analyses (Supplementary Figure 4c). When requiring complete data for the DAWBA trajectories, there was not a clear optimum number of classes: model fit improved, as indicated by the loglikelihood value, sample size adjusted Bayesian information criterion and Bootstrapped Likelihood Ratio Test from the one- to six- class solutions, but the Vuong-Lo-Mendell-Rubin Likelihood Ratio Rest indicated little improvement in model fit following the two-class solution. Given the theoretical justification for identifying more than two classes, the three- four- and five-class solutions were inspected (see Supplementary Figure 4d-f) which included similar classes to those in the model used for the primary analyses, with the exception that a child-limited class was not observed. In summary, a late-onset class was observed for both SDQ and DAWBA trajectories across varying levels of missingness, but for the DAWBA models, not all the other ‘high symptom’ classes were identified when only individuals with data across all 5-time points were included.

**References**

Angold, A., Costello, E. J., Messer, S. C., Pickles, A., Winder, F., & Silver, D. (1995). Development of a short questionnaire for use in epidemiological studies of depression in children and adolescents. *International Journal of Methods in Psychiatric Research, 5*(4), 237-249.

Angulo, M., Rooks, B. T., Gill, M., Goldstein, T., Sakolsky, D., Goldstein, B., et al. (2017). Psychometrics of the screen for adult anxiety related disorders (SCAARED)- A new scale for the assessment of DSM-5 anxiety disorders. *Psychiatry Res, 253*, 84-90.

Bellgrove, M. A., Hawi, Z., Kirley, A., Gill, M., & Robertson, I. H. (2005). Dissecting the attention deficit hyperactivity disorder (ADHD) phenotype: sustained attention, response variability and spatial attentional asymmetries in relation to dopamine transporter (DAT1) genotype. *Neuropsychologia, 43*(13), 1847-1857.

Boyd, A., Golding, J., Macleod, J., Lawlor, D. A., Fraser, A., Henderson, J., et al. (2013). Cohort Profile: the 'children of the 90s'--the index offspring of the Avon Longitudinal Study of Parents and Children. *Int J Epidemiol, 42*(1), 111-127.

Demontis, D., Walters, R. K., Martin, J., Mattheisen, M., Als, T. D., Agerbo, E., et al. (2019). Discovery of the first genome-wide significant risk loci for attention deficit/hyperactivity disorder. *Nat Genet, 51*(1), 63-75.

Euesden, J., Lewis, C. M., & O'Reilly, P. F. (2015). PRSice: Polygenic Risk Score software. *Bioinformatics, 31*(9), 1466-1468.

Fraser, A., Macdonald-Wallis, C., Tilling, K., Boyd, A., Golding, J., Davey Smith, G., et al. (2013). Cohort Profile: the Avon Longitudinal Study of Parents and Children: ALSPAC mothers cohort. *Int J Epidemiol, 42*(1), 97-110.

Goodman, R. (1997). The Strengths and Difficulties Questionnaire: a research note. *J Child Psychol Psychiatry, 38*(5), 581-586.

Goodman, R., Ford, T., Richards, H., Gatward, R., & Meltzer, H. (2000). The Development and Well-Being Assessment: description and initial validation of an integrated assessment of child and adolescent psychopathology. *J Child Psychol Psychiatry, 41*(5), 645-655.

Harris, P. A., Taylor, R., Minor, B. L., Elliott, V., Fernandez, M., O'Neal, L., et al. (2019). The REDCap consortium: Building an international community of software platform partners. *J Biomed Inform, 95*, 103208.

Harris, P. A., Taylor, R., Thielke, R., Payne, J., Gonzalez, N., & Conde, J. G. (2009). Research electronic data capture (REDCap)--a metadata-driven methodology and workflow process for providing translational research informatics support. *J Biomed Inform, 42*(2), 377-381.

Hoekstra, R. A., Vinkhuyzen, A. A., Wheelwright, S., Bartels, M., Boomsma, D. I., Baron-Cohen, S., et al. (2011). The construction and validation of an abridged version of the autism-spectrum quotient (AQ-Short). *J Autism Dev Disord, 41*(5), 589-596.

Hyde, C. L., Nagle, M. W., Tian, C., Chen, X., Paciga, S. A., Wendland, J. R., et al. (2016). Identification of 15 genetic loci associated with risk of major depression in individuals of European descent. *Nat Genet, 48*(9), 1031-1036.

Metzler-Baddeley, C., Caeyenberghs, K., Foley, S., & Jones, D. K. (2016). Task complexity and location specific changes of cortical thickness in executive and salience networks after working memory training. *NeuroImage, 130*, 48-62.

Muthén, L. K., & Muthén, B. O. (1998-2012). *Mplus User's Guide* (Seventh ed.). Los Angeles, CA: Muthén & Muthén.

Northstone, K., Lewcock, M., Groom, A., Boyd, A., Macleod, J., Timpson, N., et al. (2019). The Avon Longitudinal Study of Parents and Children (ALSPAC): an update on the enrolled sample of index children in 2019. *Wellcome open research, 4*, 51-51.

Robertson, I. H., Ward, T., Ridgeway, V., & Nimmo-Smith, I. (1996). The structure of normal human attention: The Test of Everyday Attention. *Journal of the International Neuropsychological Society, 2*(6), 525-534.

Sayon-Orea, C., Moreno-Iribas, C., Delfrade, J., Sanchez-Echenique, M., Amiano, P., Ardanaz, E., et al. (2020). Inverse-probability weighting and multiple imputation for evaluating selection bias in the estimation of childhood obesity prevalence using data from electronic health records. *BMC Medical Informatics and Decision Making, 20*(1), 9.

Seaman, S. R., & White, I. R. (2013). Review of inverse probability weighting for dealing with missing data. *Stat Methods Med Res, 22*(3), 278-295.

Seaman, S. R., White, I. R., Copas, A. J., & Li, L. (2012). Combining multiple imputation and inverse-probability weighting. *Biometrics, 68*(1), 129-137.

Sibley, M. H., Arnold, L. E., Swanson, J. M., Hechtman, L. T., Kennedy, T. M., Owens, E., et al. (2021). Variable Patterns of Remission From ADHD in the Multimodal Treatment Study of ADHD. *American Journal of Psychiatry*, appi.ajp.2021.21010032.

Skuse, D. H., Mandy, W. P., & Scourfield, J. (2005). Measuring autistic traits: heritability, reliability and validity of the Social and Communication Disorders Checklist. *Br J Psychiatry, 187*(6), 568-572.

Stroop, J. R. (1935). Studies of interference in serial verbal reactions. *Journal of experimental psychology, 18*(6), 643.

Thabrew, H., Stasiak, K., Bavin, L. M., Frampton, C., & Merry, S. (2018). Validation of the Mood and Feelings Questionnaire (MFQ) and Short Mood and Feelings Questionnaire (SMFQ) in New Zealand help-seeking adolescents. *Int J Methods Psychiatr Res, 27*(3), e1610.

von Hippel, P. T. (2018). How Many Imputations Do You Need? A Two-stage Calculation Using a Quadratic Rule. *Sociological Methods & Research*, doi: 10.1177/0049124117747303.

White, I. R., Royston, P., & Wood, A. M. (2011). Multiple imputation using chained equations: Issues and guidance for practice. *Stat Med, 30*(4), 377-399.

Wray, N. R., Ripke, S., Mattheisen, M., Trzaskowski, M., Byrne, E. M., Abdellaoui, A., et al. (2018). Genome-wide association analyses identify 44 risk variants and refine the genetic architecture of major depression. *Nat Genet, 50*(5), 668-681.

| **Supplementary Table 1.** ADHD groupings for cut-point based definitions | | | |
| --- | --- | --- | --- |
|  |  | Symptoms in adolescence and/or adulthood | |
|  |  | Not high (<8 at age 17 and <5 for self-report /  <4 for parent-report at age 25) | High (≥8 at age 17 or ≥5 for self-report /  ≥4 for parent-report at age 25) |
| Symptoms in childhood | Low (<6 at age 7, 8, 9 and 12 years) | Low symptoms | Late-onset ADHD |
|  | Subthreshold (score ≥6 at age 7, 8, 9 or 12 years  and <8 at age 7, 8, 9 and 12 years) | Low symptoms | Subthreshold late-onset ADHD |
|  | High (score ≥8 at age 7, 8, 9 or 12 years) | Child-limited ADHD | Child-onset persistent ADHD |

| **Supplementary Table 2**. Associations for ADHD risk factors with late-onset and child-onset persistent compared to low ADHD symptoms | | | | | | | | |
| --- | --- | --- | --- | --- | --- | --- | --- | --- |
|  | SDQ cut-points: adult self-reports | | SDQ cut-points: parent-reports | | Latent trajectories: SDQ | | Latent trajectories: DAWBA | |
|  | Late-onset | Child-onset persistent | Late-onset | Child-onset persistent | Late-onset | Child-onset persistent | Late-onset | Child-onset persistent |
| Male sex | OR=1.19 (0.98-1.46), p=0.08 | OR=2.94 (2.14-4.05), p<0.001 | OR=0.98 (0.73-1.31), p=0.87 | OR=3.18 (2.26-4.47), p<0.001 | OR=1.08 (0.82-1.43), p=0.59 | OR=4.93 (3.66-6.65), p<0.001 | OR=1.55 (1.16-2.09), p=0.003 | OR=3.48 (2.35-5.14), p<0.001 |
| ADHD PRS | OR=1.02 (0.92-1.13), p=0.73 | OR=1.45 (1.22-1.72), p<0.001 | OR=1.27 (1.08-1.50), p=0.004 | OR=1.54 (1.28-1.84), p<0.001 | OR=1.32 (1.12-1.56), p=0.001 | 1.44 (1.23-1.69), p<0.001 | OR=1.35 (1.13-1.62), p=0.001 | OR=1.51 (1.19-1.92), p=0.001 |
| Preterm birth | OR=0.72 (0.41-1.26), p=0.25 | OR=1.41 (0.72-2.72), p=0.31 | OR=1.03 (0.50-2.13), p=0.93 | OR=1.92 (1.03-3.60), p=0.04 | OR=1.98 (1.13-3.49), p=0.02 | OR=2.39 (1.58-3.63), p<0.001 | OR=0.84 (0.37-1.94), p=0.69 | OR=1.90 (0.99-3.64), p=0.05 |
| Low birth weight | OR=1.12 (0.68-1.84), p=0.65 | OR=1.08 (0.50-2.35), p=0.85 | OR=0.52 (0.19-1.44), p=0.21 | OR=1.38 (0.68-2.80), p=0.38 | OR=1.46 (0.75-2.82), p=0.26 | OR=1.46 (1.00-2.13), p=0.05 | OR=1.08 (0.50-2.34), p=0.85 | OR=1.96 (0.99-3.88), p=0.05 |
| SDQ = Strengths and Difficulties Questionnaire, DAWBA = Development and Well-Being Assessment. 95% confidence intervals in parentheses. Multinomial odds ratios based on ADHD groups as the outcome. Cut-point based analyses using multiple imputation with inverse probability weighting, trajectory definitions using full information maximum likelihood to derive trajectories and listwise deletion for associations with other variables. PRS were standardized to mean=0 SD=1. | | | | | | | | |

| **Supplementary Table 3**. Associations for cognitive tasks with late-onset and child-onset persistent compared to low ADHD symptoms | | | | | | | | |
| --- | --- | --- | --- | --- | --- | --- | --- | --- |
|  | SDQ cut-points: adult self-reports | | SDQ cut-points: parent-reports | | Latent trajectories: SDQ | | Latent trajectories: DAWBA | |
|  | Late-onset | Child-onset persistent | Late-onset | Child-onset persistent | Late-onset | Child-onset persistent | Late-onset | Child-onset persistent |
| Child sustained attention | OR=0.96 (0.86-1.06), p=0.39 | OR=0.77 (0.67-0.90), p=0.001 | OR=0.98 (0.84-1.13), p=0.76 | OR=0.78 (0.67-0.91), p=0.002 | OR=1.02 (0.75-1.39), p=0.90 | OR=0.69 (0.61-0.79), p<0.001 | OR=0.76 (0.66-0.88), p<0.001 | OR=0.73 (0.60-0.89), p=0.002 |
| Child response inhibition | OR=0.99 (0.89-1.10), p=0.86 | OR=0.81 (0.70-0.95), p=0.01 | OR=0.87 (0.75-1.01), p=0.06 | OR=0.79 (0.68-0.91), p=0.001 | OR=0.91 (0.70-1.19), p=0.49 | OR=0.71 (0.62-0.80), p<0.001 | OR=0.80 (0.66-0.96), p=0.02 | OR=0.66 (0.57-0.78), p<0.001 |
| Adult sustained attention | OR=0.87 (0.77-0.98), p=0.03 | OR=0.69 (0.54-0.87), p=0.002 | OR=0.78 (0.64-0.96), p=0.02 | OR=0.69 (0.54-0.90), p=0.01 | OR=0.59 (0.34-1.02), p=0.06 | OR=0.50 (0.24-1.03), p=0.06 | OR=0.87 (0.58-1.32), p=0.52 | OR=1.51 (0.64-3.58), p=0.35 |
| Adult response inhibition | OR=0.91 (0.79-1.06), p=0.24 | OR=0.68 (0.55-0.85), p=0.001 | OR=0.79 (0.64-0.97), p=0.02 | OR=0.65 (0.51-0.83), p=0.001 | OR=0.69 (0.48-1.00), p=0.05 | OR=0.47 (0.23-0.94), p=0.03 | OR=0.73 (0.47-1.11), p=0.14 | OR=1.82 (0.22-14.82), p=0.57 |
| SDQ = Strengths and Difficulties Questionnaire, DAWBA = Development and Well-Being Assessment. 95% confidence intervals in parentheses. Multinomial odds ratios based on ADHD groups as the outcome. Cut-point based analyses using multiple imputation with inverse probability weighting, trajectory definitions using full information maximum likelihood to derive trajectories and listwise deletion for associations with other variables. Cognitive scores were standardized to mean=0 SD=1 to aid interpretation. | | | | | | | | |

| **Supplementary Table 4**. Associations for childhood resources with late-onset and child-onset persistent compared to low ADHD symptoms | | | | | | | | |
| --- | --- | --- | --- | --- | --- | --- | --- | --- |
|  | SDQ cut-points: adult self-reports | | SDQ cut-points: parent-reports | | Latent trajectories: SDQ | | Latent trajectories: DAWBA | |
|  | Late-onset | Child-onset persistent | Late-onset | Child-onset persistent | Late-onset | Child-onset persistent | Late-onset | Child-onset persistent |
| Verbal ability | OR=1.00 (1.00-1.01), p=0.41 | OR=0.97 (0.96-0.98), p<0.001 | OR=0.98 (0.97-0.99), p=0.001 | OR=0.96 (0.95-0.97), p<0.001 | OR=0.98 (0.97-1.00), p=0.004 | OR=0.96 (0.95-0.97), p<0.001 | OR=0.97 (0.96-0.98), p<0.001 | OR=0.95 (0.94-0.97), p<0.001 |
| Reading ability | OR=1.01 (1.00-1.02), p=0.17 | OR=0.93 (0.92-0.95), p<0.001 | OR=0.98 (0.96-1.00), p=0.02 | OR=0.91 (0.90-0.93), p<0.001 | OR=0.97 (0.95-0.98), p<0.001 | OR=0.89 (0.88-0.91), p<0.001 | OR=0.96 (0.94-0.98), p<0.001 | OR=0.89 (0.87-0.91), p<0.001 |
| Family income | OR=1.00 (0.95-1.05), p=0.93 | OR=0.88 (0.83-0.93), p<0.001 | OR=0.94 (0.89-1.00), p=0.07 | OR=0.85 (0.80-0.91), p<0.001 | OR=0.98 (0.93-1.04), p=0.58 | OR=0.50 (0.24-1.03), p=0.06 | OR=0.90 (0.85-0.96), p=0.001 | OR=0.82 (0.76-0.89), p<0.001 |
| Maternal education | OR=1.04 (0.96-1.13), p=0.32 | OR=0.82 (0.72-0.93), p=0.002 | OR=0.91 (0.80-1.02), p=0.11 | OR=0.81 (0.72-0.93), p=0.002 | OR=1.12 (1.00-1.24), p=0.05 | OR=0.74 (0.67-0.81), p<0.001 | OR=0.96 (0.85-1.08), p=0.53 | OR=0.77 (0.67-0.89), p<0.001 |
| SDQ = Strengths and Difficulties Questionnaire, DAWBA = Development and Well-Being Assessment. 95% confidence intervals in parentheses. Multinomial odds ratios based on ADHD groups as the outcome. Verbal and reading ability observed scores 46-155 and 0-50 respectively, family income and maternal education assessed on ordinal scales (1-10 and 1-5 respectively). | | | | | | | | |

| **Supplementary Table 5**. Associations for depression risk factors with late-onset and child-onset persistent compared to low ADHD symptoms | | | | | | | | |
| --- | --- | --- | --- | --- | --- | --- | --- | --- |
|  | SDQ cut-points: adult self-reports | | SDQ cut-points: parent-reports | | Latent trajectories: SDQ | | Latent trajectories: DAWBA | |
|  | Late-onset | Child-onset persistent | Late-onset | Child-onset persistent | Late-onset | Child-onset persistent | Late-onset | Child-onset persistent |
| Depression PRS | OR=1.08 (0.97-1.20), p=0.16 | OR=1.20 (1.00-1.43), =0.05 | OR=1.03 (0.87-1.20), p=0.75 | OR=1.05 (0.88-1.26), p=0.55 | OR=1.11 (0.96-1.28), p=0.16 | OR=1.07 (0.94-1.24), p=0.312 | OR=1.01 (0.87-1.18), p=0.87 | OR=1.10 (0.88-1.37), p=0.39 |
| History of maternal depression | OR=1.28 (0.86-1.92), p=0.22 | OR=1.40 (0.77-2.56), p=0.27 | OR=2.48 (1.57-3.94), p<0.001 | OR=1.51 (0.82-2.79), p=0.19 | OR=1.93 (1.20-3.09), p=0.01 | OR=3.30 (2.39-4.56), p<0.001 | OR=3.42 (2.27-5.16), p<0.001 | OR=2.71 (1.63-4.52), p<0.001 |
| SDQ = Strengths and Difficulties Questionnaire, DAWBA = Development and Well-Being Assessment. 95% confidence intervals in parentheses. Multinomial odds ratios based on ADHD groups as the outcome. PRS were standardized to mean=0 SD=1. | | | | | | | | |

| **Supplementary Table 6.** Associations between analysis variables and missingness (N=4224) for cut-point based analyses | | | | | | |  |
| --- | --- | --- | --- | --- | --- | --- | --- |
|  | Available data | | Association with missing ADHD symptom data^*^ | | Association with  missing additional variable data^*^ | |  |
|  | % | N | OR | (95% CI) | OR | (95% CI) | |
| ***ADHD*** |  |  |  |  |  |  | |
| Age 7 SDQ score | 90% | (3812) | 1.07 | (1.04-1.10) | 1.07 | (1.01-1.13) | |
| Age 8 SDQ score | 90% | (3811) | 1.08 | (1.06-1.11) | 1.09 | (1.03-1.15) | |
| Age 9 SDQ score | 95% | (3996) | 1.09 | (1.06-1.12) | 1.08 | (1.02-1.14) | |
| Age 12 SDQ score | 93% | (3936) | 1.12 | (1.09-1.15) | 1.12 | (1.06-1.19) | |
| Age 17 SDQ score | 100% | (4224) | 1.09 | (1.06-1.13) | 1.13 | (1.05-1.20) | |
| Age 25 SDQ score: parent-rated | 84% | (3557) | 1.15 | (1.10-1.19) | 1.19 | (1.10-1.29) | |
| Age 25 SDQ score: self-rated | 70% | (2955) | 1.03 | (1.00-1.08) | 1.02 | (0.96-1.08) | |
| ADHD medication | 68% | (2868) | 0.63 | (0.30-1.30) | 0.79 | (0.30-2.03) | |
| ***ADHD risk factors*** |  |  |  |  |  |  | |
| ADHD polygenic score | 75% | (3175) | 1.06 | (0.99-1.14) | 1.23 | (1.09-1.38) | |
| Preterm birth | 95% | (4019) | 1.23 | (0.90-1.66) | 0.60 | (0.37-0.95) | |
| Low birth weight | 94% | (3974) | 0.90 | (0.66-1.23) | 0.84 | (0.49-1.44) | |
| ***Cognitive tasks*** |  |  |  |  |  |  | |
| Child sustained attention | 81% | (3442) | 0.94 | (0.88-1.01) | 0.94 | (0.84-1.05) | |
| Child response inhibition | 82% | (3453) | 0.94 | (0.88-1.01) | 0.86 | (0.76-0.97) | |
| Adult sustained attention | 23% | (980) | 0.97 | (0.85-1.11) | 0.97 | (0.85-1.11) | |
| Adult response inhibition | 25% | (1058) | 0.89 | (0.78-1.01) | 0.84 | (0.73-0.97) | |
| ***Childhood resources*** |  |  |  |  |  |  | |
| Child verbal IQ | 85% | (3608) | 0.99 | (0.98-0.99) | 0.98 | (0.98-0.98) | |
| Reading ability | 87% | (3690) | 0.98 | (0.97-0.98) | 0.96 | (0.95-0.98) | |
| Family income | 82% | (3479) | 0.92 | (0.89-0.94) | 0.90 | (0.85-0.95) | |
| Maternal education | 95% | (4000) | 0.84 | (0.80-0.89) | 0.71 | (0.64-0.80) | |
| ***Depression risk factors*** |  |  |  |  |  |  | |
| Depression polygenic score | 75% | (3175) | 1.04 | (0.97-1.12) | 1.07 | (0.95-1.21) | |
| Maternal depression | 96% | (3994) | 1.56 | (1.19-2.05) | 1.08 | (0.65-1.79) | |
| ***Other psychopathology*** |  |  |  |  |  |  | |
| Child social-communication problems | 90% | (3814) | 1.43 | (1.09-1.87) | 1.50 | (0.85-2.66) | |
| Child emotional problems | 93% | (3938) | 1.13 | (0.88-1.46) | 1.36 | (0.80-2.32) | |
| Child depression symptoms | 85% | (3595) | 1.15 | (0.85-1.56) | 1.18 | (0.66-2.11) | |
| Adult autism symptoms: parent-rated | 84% | (3536) | 1.24 | (0.95-1.64) | 1.31 | (0.76-2.24) | |
| Adult autism symptoms: self-rated | 66% | (2804) | 1.04 | (0.83-1.29) | 0.68 | (0.50-0.93) | |
| Adult anxiety symptoms | 69% | (2930) | 1.28 | (1.10-1.49) | 0.98 | (0.77-1.25) | |
| Adult depressive symptoms | 67% | (2824) | 1.45 | (1.19-1.75) | 0.98 | (0.72-1.33) | |
| ^*^Partial vs complete SDQ data. SDQ = Strengths and Difficulties Questionnaire. Device type included as a covariate for adult cognitive tasks regressions. PRS and cognitive tasks standardized to mean=0 SD=1. Family income and maternal education = ordinal scales (possible range 1-10 and 1-5 respectively). | | | | | | |  |

| **Supplementary Table 7.** Associations between variables included in the inverse probability weight for cut-point based analyses: association with exclusion from the primary sample (missing ADHD data) | | |
| --- | --- | --- |
|  | Initial multivariable analyses | Final multivariable analysis from IPW model |
| Enrolled in original ALSPAC sample | OR=0.54, 95% CI=0.45-0.64 | OR=0.43, 95% CI=0.36-0.52 |
| Male sex | OR=1.43, 95% CI=1.33-1.53 | OR=1.51 95% CI=1.40-1.64 |
| Home ownership | OR=0.36, 95% CI=0.32-0.40 | OR=0.56, 95% CI=0.50-0.63 |
| Low birth weight | OR=1.28, 95% CI=1.07-1.53 | - |
| Preterm birth | OR=1.17, 95% CI=0.98-1.39 | - |
| History of maternal depression | OR=1.52, 95% CI=1.31-1.76 | OR=1.33, 95% CI=1.13-1.56 |
| Maternal age at birth (years) | OR=0.90, 95% CI=0.89-0.91 | OR=0.92, 95% CI=0.91-0.93 |
| Maternal education (1-5 scale) | OR=0.57, 95% CI=0.55-0.59 | OR=0.66, 95% CI=0.64-0.68 |
| Parity | OR=1.09, 95% CI=1.05-1.14 | OR=1.21, 95% CI=1.15-1.27 |

| **Supplementary Table 8**. Model fit indices for growth mixture models for the Strengths and Difficulties Questionnaire (SDQ) and Development and Well-Being Assessment (DAWBA) | | | | | | | |
| --- | --- | --- | --- | --- | --- | --- | --- |
|  | LL | Free parameters | ssaBIC | Smallest class | Entropy | VLMR-LRT  p value | BLRT  p value |
| *SDQ* |  |  |  |  |  |  |  |
| 1 class | -110625.46 | 14 | 221335.04 |  |  |  |  |
| 2 classes | -109765.53 | 18 | 219639.21 | 17.14% (N=1673) | 0.76 | <0.0001 | <0.0001 |
| 3 classes | -109468.55 | 22 | 219069.28 | 6.95% (N=678) | 0.70 | 0.10 | <0.0001 |
| 4 classes^*^ | -109138.71 | 26 | 218433.64 | 5.31% (N=518) | 0.70 | <0.0001 | <0.0001 |
| 5 classes | -109035.64 | 30 | 218251.53 | 5.06% (N=494) | 0.69 | 0.05 | <0.0001 |
| *DAWBA* |  |  |  |  |  |  |  |
| 1 class | -88763.60 | 11 | 177591.28 |  |  |  |  |
| 2 classes | -86431.97 | 15 | 172951.32 | 8.89% (N=351) | 0.95 | <0.0001 | <0.0001 |
| 3 classes | -85279.39 | 19 | 170669.47 | 5.82% (N=233) | 0.93 | 0.0005 | <0.0001 |
| 4 classes | -84366.34 | 23 | 168866.67 | 2.74% (N=103) | 0.91 | 0.0003 | <0.0001 |
| 5 classes^*^ | -83793.13 | 27 | 167743.56 | 1.91% (N=155) | 0.92 | 0.03 | <0.0001 |
| 6 classes | -83331.95 | 31 | 166844.50 | 1.39% (N=113) | 0.91 | 0.13 | <0.0001 |
| LL=Loglikelihood; ssa=sample size adjusted; BIC=Bayesian Information Criteria; VLMR-LRT=Vuong-Lo-Mendell-Rubin Likelihood Ratio Rest; BLRT=Bootstrapped Likelihood Ratio Test. ^*^Final model. | | | | | | | |

| **Table 9.** Prevalence of ADHD groups for cut-point based analyses using different approaches to missing data | | | | | | | |
| --- | --- | --- | --- | --- | --- | --- | --- |
|  | Childhood symptoms | Late-adolescent/young-adult symptoms | Low | Child-limited | Child-onset persistent | Late-onset | Subthreshold late-onset |
| Self-reports^#^ |  |  |  |  |  |  |  |
| Primary analyses: IPW/MI | 11.7% | 26.3% | 67.9% | 5.8% | 5.9% | 14.4% | 6.0% |
| Complete cases | 8.3% | 24.9% | 70.7% | 4.4% | 4.0% | 16.3% | 4.6% |
| Complete cases with IPW | 9.0% | 25.8% | 69.6% | 4.6% | 4.4% | 16.4% | 5.0% |
| Parent-reports |  |  |  |  |  |  |  |
| Primary analyses: IPW/MI | 11.7% | 15.0% | 78.9% | 6.2% | 5.6% | 5.3% | 4.1% |
| Complete cases | 8.3% | 9.7% | 84.8% | 5.5% | 2.8% | 4.4% | 2.4% |
| Complete cases with IPW | 9.0% | 10.2% | 83.9% | 5.9% | 3.1% | 4.5% | 2.6% |
| ^#^Self-reports in adulthood, parent-reports for prior assessments. IPW = inverse probability weighting, MI = multiple imputation. | | | | | | | |

| **Supplementary Table 10**. Model fit indices for growth mixture models for the Strengths and Difficulties Questionnaire with varying levels of missingness | | | | | | | |
| --- | --- | --- | --- | --- | --- | --- | --- |
|  | LL | Free parameters | ssaBIC | Smallest class | Entropy | VLMR-LRT  p value | BLRT  p value |
| *1+ data-points: N=11,137* | | | | | | | |
| 1 class | -113818.31 | 14 | 227722.59 |  |  |  |  |
| 2 classes | -112937.43 | 18 | 225985.39 | 17.55% (N=1954) | 0.72 | <0.0001 | <0.0001 |
| 3 classes | -112642.88 | 22 | 225420.84 | 7.05% (N=785) | 0.67 | 0.37 | <0.0001 |
| 4 classes | -112310.72 | 26 | 224781.09 | 5.59% (N=623) | 0.66 | <0.0001 | <0.0001 |
| 5 classes | -112207.73 | 30 | 224599.66 | 5.33% (N=594) | 0.65 | 0.06 | <0.0001 |
| *2+ data-points: N=9,764 (primary analyses)* | | | | | | | |
| 1 class | -110625.46 | 14 | 221335.04 |  |  |  |  |
| 2 classes | -109765.53 | 18 | 219639.21 | 17.14% (N=1673) | 0.76 | <0.0001 | <0.0001 |
| 3 classes | -109468.55 | 22 | 219069.28 | 6.95% (N=678) | 0.70 | 0.10 | <0.0001 |
| 4 classes^*^ | -109138.71 | 26 | 218433.64 | 5.31% (N=518) | 0.70 | <0.0001 | <0.0001 |
| 5 classes | -109035.64 | 30 | 218251.53 | 5.06% (N=494) | 0.69 | 0.05 | <0.0001 |
| *8 data-points: N=2,737 (complete cases)* | | | | | | | |
| 1 class | -41667.98 | 14 | 83402.28 |  |  |  |  |
| 2 classes | -41303.12 | 18 | 82691.50 | 17.46% (N=478) | 0.79 | <0.0001 | <0.0001 |
| 3 classes | -41110.85 | 22 | 82325.92 | 6.61% (N=181) | 0.83 | 0.03 | <0.0001 |
| 4 classes | -40964.59 | 26 | 82052.35 | 4.40% (N=120) | 0.83 | 0.09 | <0.0001 |
| 5 classes | -40904.14 | 30 | 81950.41 | 2.07% (N=57) | 0.82 | 0.24 | <0.0001 |
| LL=Loglikelihood; ssa=sample size adjusted; BIC=Bayesian Information Criteria; VLMR-LRT=Vuong-Lo-Mendell-Rubin Likelihood Ratio Rest; BLRT=Bootstrapped Likelihood Ratio Test. ^*^Final model. | | | | | | | |

| **Supplementary Table 11**. Model fit indices for growth mixture models for Development and Well-Being Assessment with varying levels of missingness | | | | | | | |
| --- | --- | --- | --- | --- | --- | --- | --- |
|  | LL | Free parameters | ssaBIC | Smallest class | Entropy | VLMR-LRT  p value | BLRT  p value |
| *1+ data-points: N=9,803* | | | | | | | |
| 1 class | -94520.58 | 11 | 189107.30 |  |  |  |  |
| 2 classes | -91919.79 | 15 | 183929.77 | 8.98% (N=880) | 0.94 | <0.0001 | <0.0001 |
| 3 classes | -90713.71 | 19 | 181541.67 | 3.74% (N=367) | 0.90 | 0.0007 | <0.0001 |
| 4 classes | -89741.08 | 23 | 179620.46 | 3.10% (N=304) | 0.89 | 0.0002 | <0.0001 |
| 5 classes | -89086.70 | 27 | 178335.74 | 2.03% (N=199) | 0.89 | 0.32 | <0.0001 |
| 6 classes | -88612.88 | 31 | 177412.15 | 2.26% (N=222) | 0.87 | 0.42 | <0.0001 |
| *2+ data-points: N=8,132 (primary analyses)* | | | | | | | |
| 1 class | -88763.60 | 11 | 177591.28 |  |  |  |  |
| 2 classes | -86431.97 | 15 | 172951.32 | 8.89% (N=351) | 0.95 | <0.0001 | <0.0001 |
| 3 classes | -85279.39 | 19 | 170669.47 | 5.82% (N=233) | 0.93 | 0.0005 | <0.0001 |
| 4 classes | -84366.34 | 23 | 168866.67 | 2.74% (N=103) | 0.91 | 0.0003 | <0.0001 |
| 5 classes^*^ | -83793.13 | 27 | 167743.56 | 1.91% (N=155) | 0.92 | 0.03 | <0.0001 |
| 6 classes | -83331.95 | 31 | 166844.50 | 1.39% (N=113) | 0.91 | 0.13 | <0.0001 |
| *5 data-points: N=2,364 (complete cases)* | | | | | | | |
| 1 class | -33473.46 | 11 | 66997.41 |  |  |  |  |
| 2 classes | -32585.68 | 15 | 65240.23 | 7.33% (N=173) | 0.97 | 0.0001 | <0.0001 |
| 3 classes | -32007.56 | 19 | 64102.34 | 3.90% (N=92) | 0.98 | 0.06 | <0.0001 |
| 4 classes | -31735.09 | 23 | 63575.77 | 1.90% (N=45) | 0.98 | 0.13 | <0.0001 |
| 5 classes | -31413.58 | 27 | 62951.11 | 1.27% (N=30) | 0.96 | 0.27 | <0.0001 |
| 6 classes | -31194.49 | 31 | 62531.30 | 0.09% (N=21) | 0.97 | 0.43 | <0.0001 |
| LL=Loglikelihood; ssa=sample size adjusted; BIC=Bayesian Information Criteria; VLMR-LRT=Vuong-Lo-Mendell-Rubin Likelihood Ratio Rest; BLRT=Bootstrapped Likelihood Ratio Test. ^*^Final model. | | | | | | | |

| **Supplementary Figure 1**. Proportion of individuals with persistent, remitted and fluctuating ADHD symptoms | |
| --- | --- |
| a) Self-rated adult symptoms | b) Parent-rated adult symptoms |
| 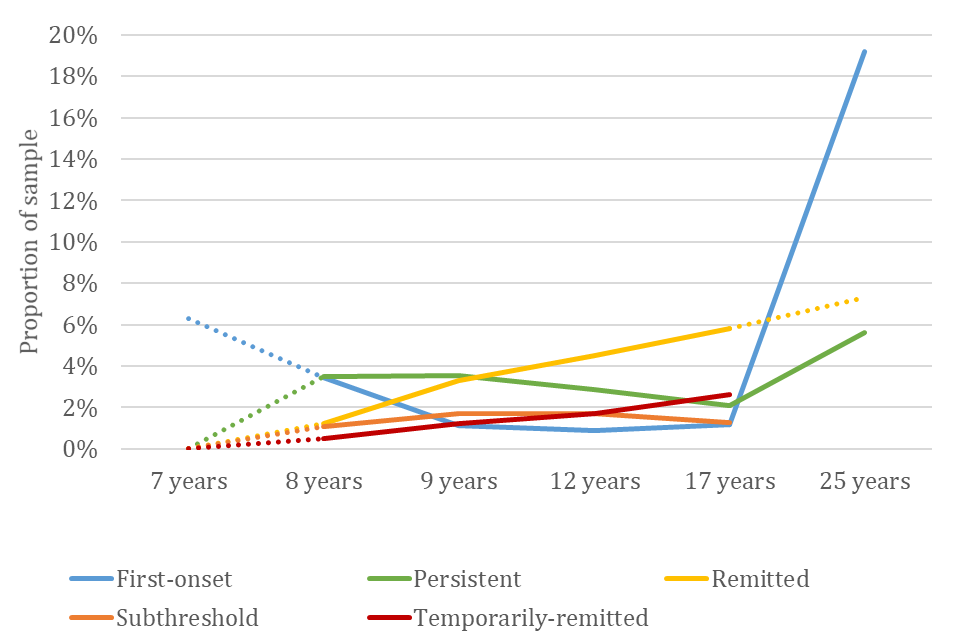 | 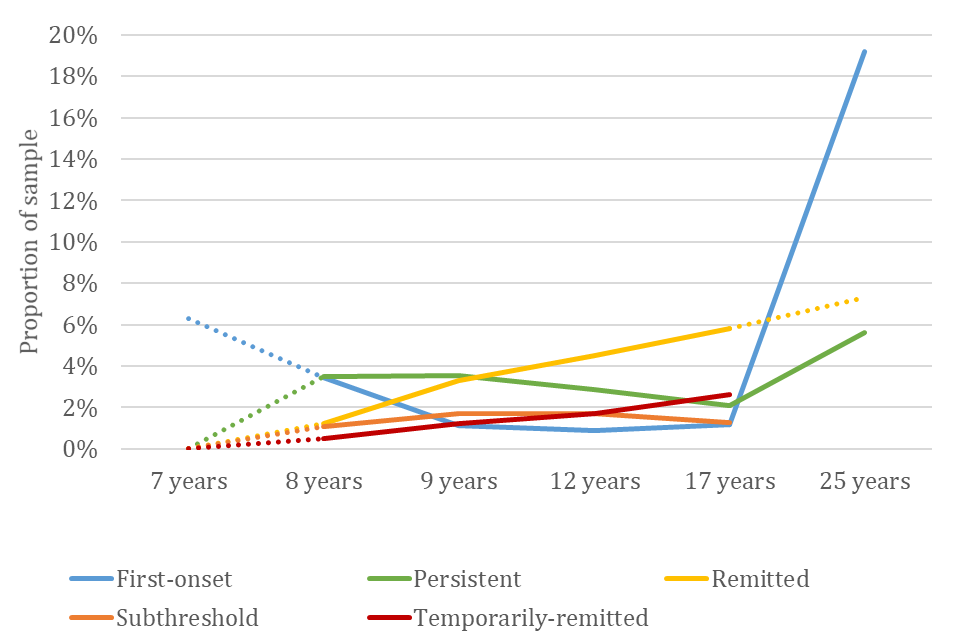 |
| ADHD symptoms measures using the Strengths & Difficulties Questionnaire. | |

| **Supplementary Figure 2**. Autism, anxiety and depression symptoms by ADHD group |
| --- |
| a) SDQ cut-point based groups: Self-rated adult symptoms |
| **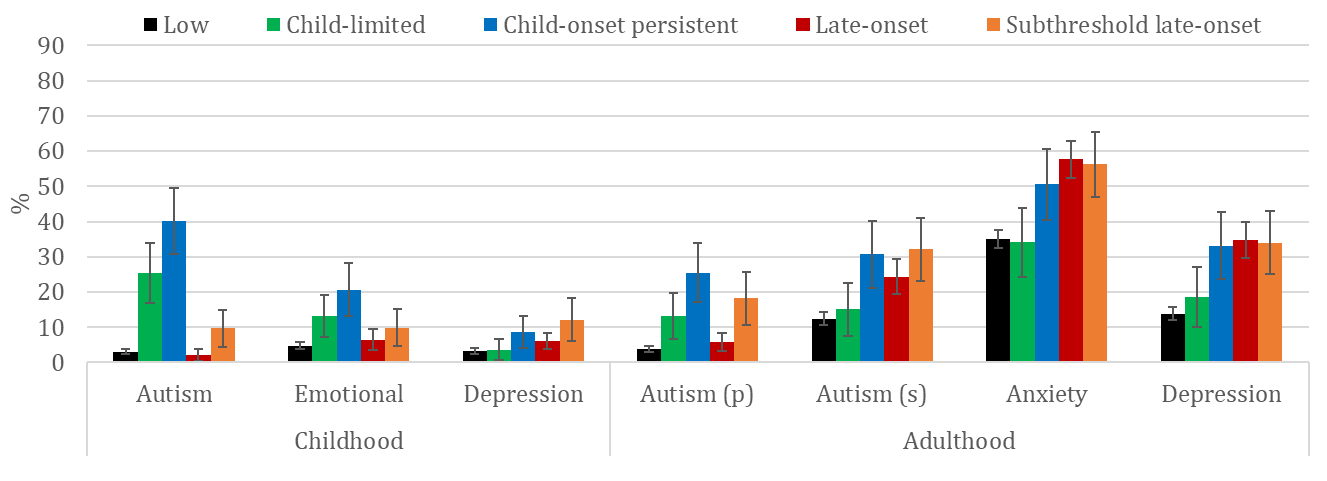** |
| b) SDQ cut-point based groups: Parent-rated adult symptoms |
| **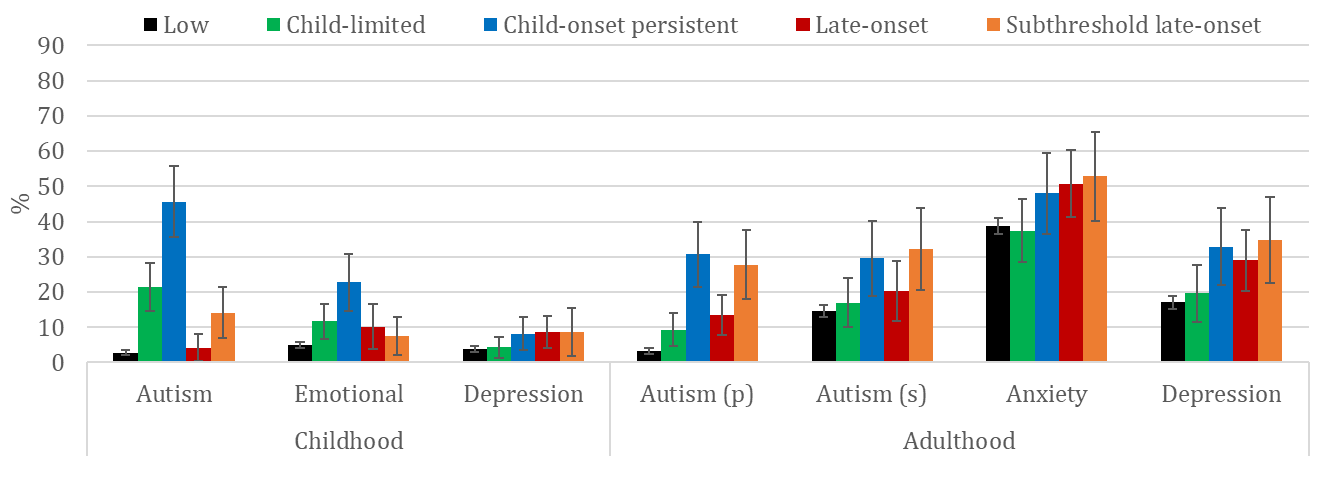** |
| c) Latent trajectory-based groups: Strengths and Difficulties Questionnaire |
| **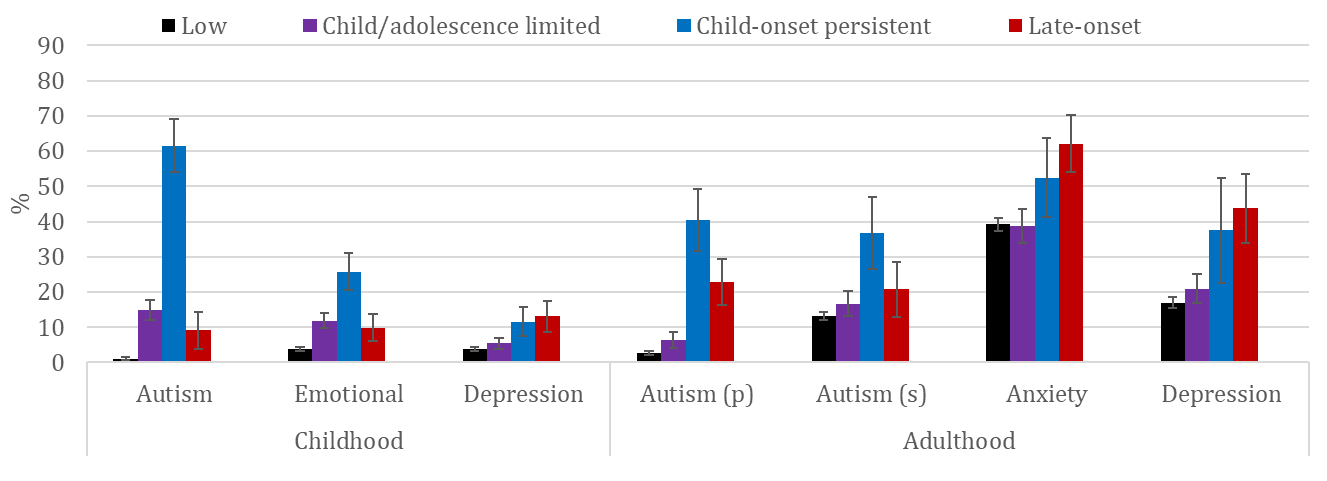** |
| d) Latent trajectory-based groups: Development and Well-Being Assessment |
| **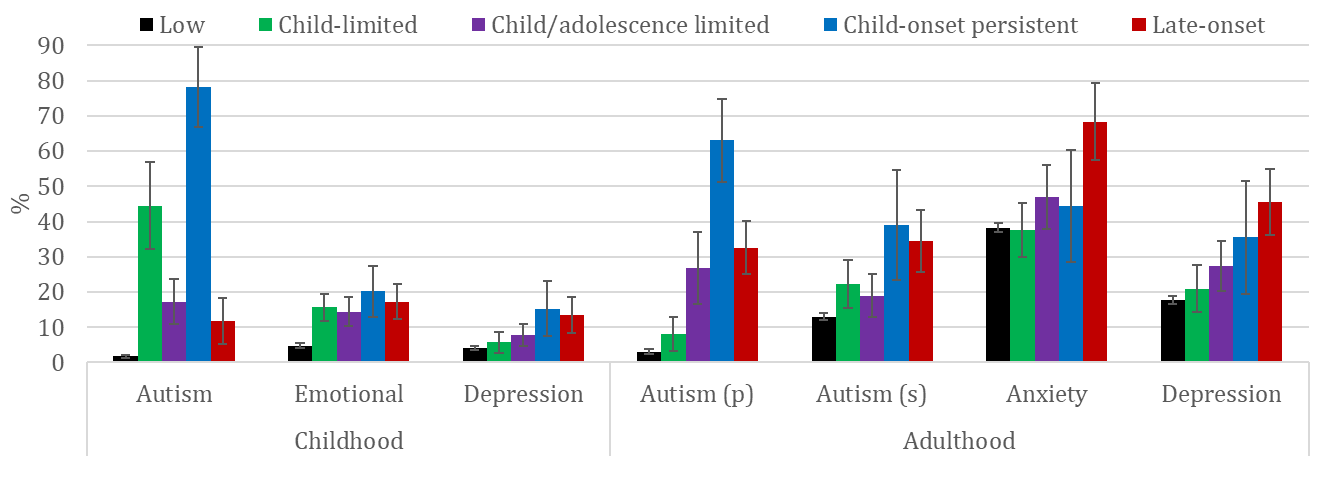** |
| p=parent-rated, s=self-rated. Measures parent-rated unless otherwise specified. Autism assessed using the Social Communication Disorders Checklist in childhood and abridged Autism-Spectrum Quotient in adulthood, anxiety symptoms using the Strengths & Difficulties Questionnaire and depression using the short Moods and Feelings Questionnaire. |
| **Supplementary** **Figure 3.** Strengths and Difficulties Questionnaire score by class |
| a) 1+ data-points (N=11,137) |
| 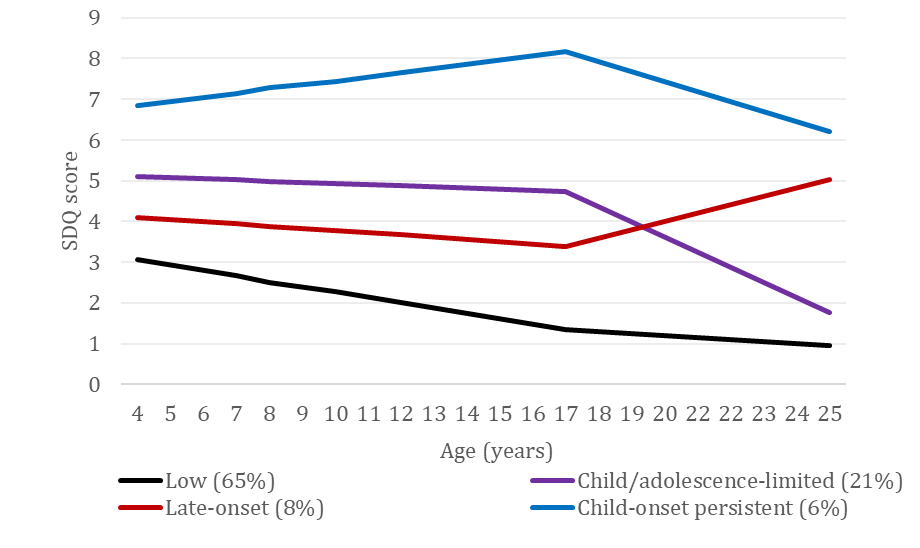 |
| b) 2+ data-points (N=9,764): primary analyses |
| 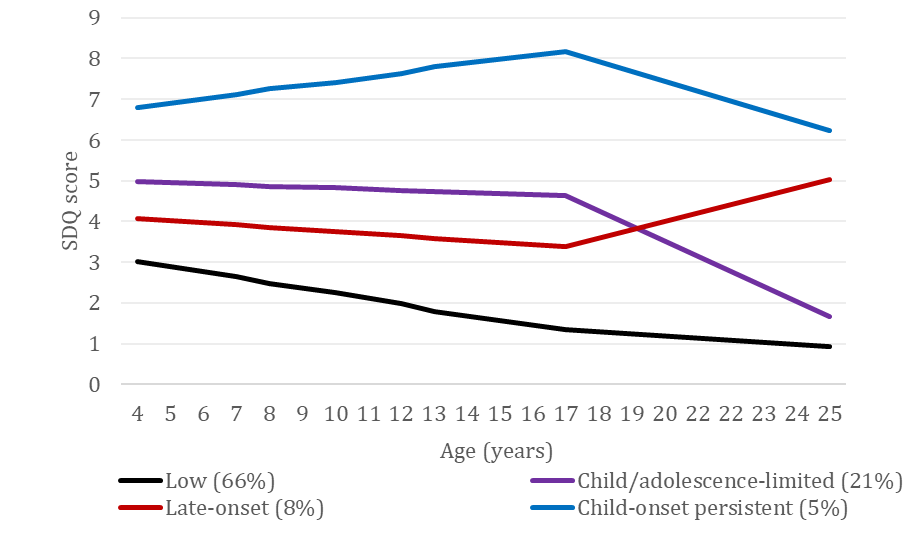 |
| c) 8 data-points (complete cases: N=2,737) |
| 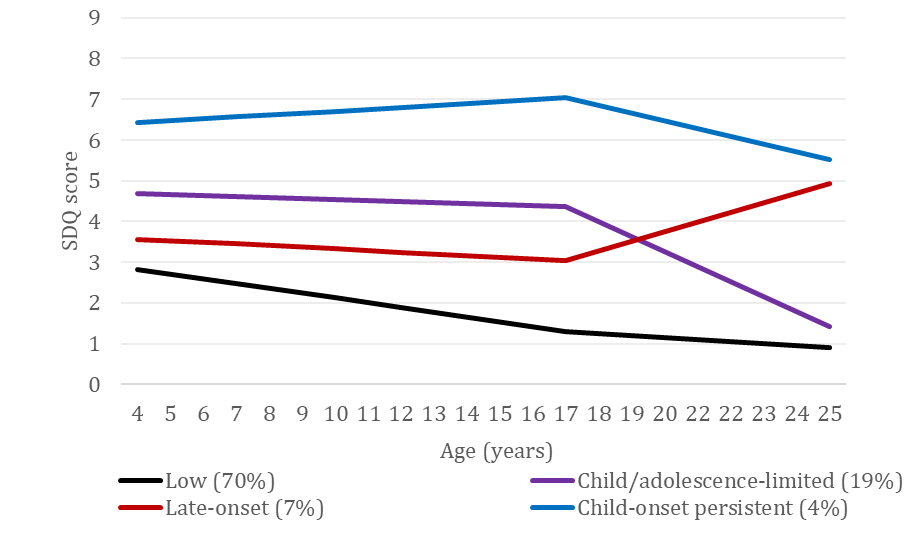 |

| **Supplementary** **Figure 4a-c.** Development and Well-Being Assessment score by class |
| --- |
| a) 1+ data-points (N=9,803): 4-class solution |
| 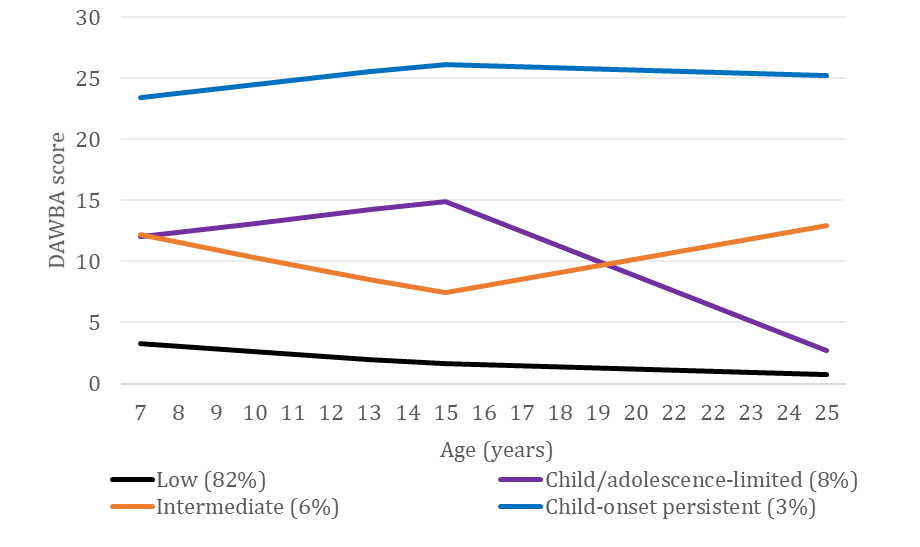 |
| b) 1+ data-points (N=9,803): 5-class solution |
| 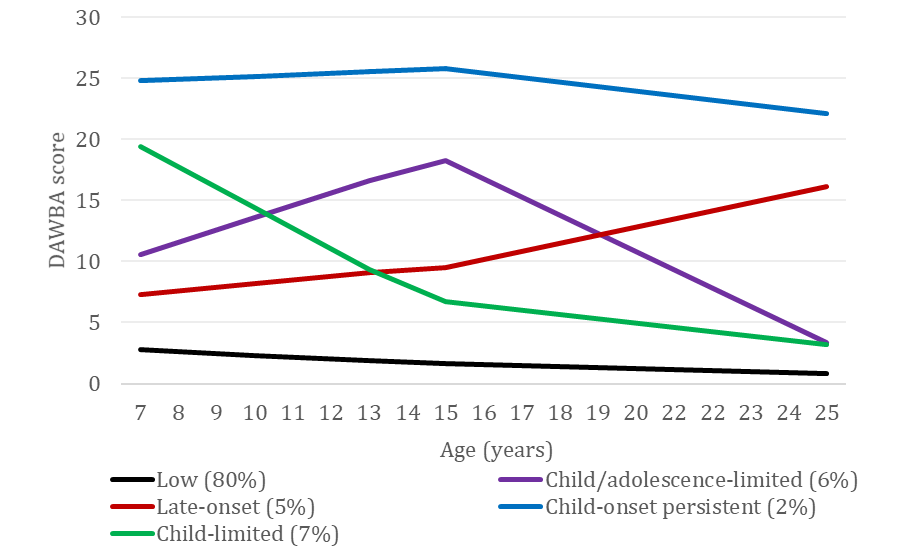 |
| c) 2+ data-points (N=8,132): primary analyses |
| 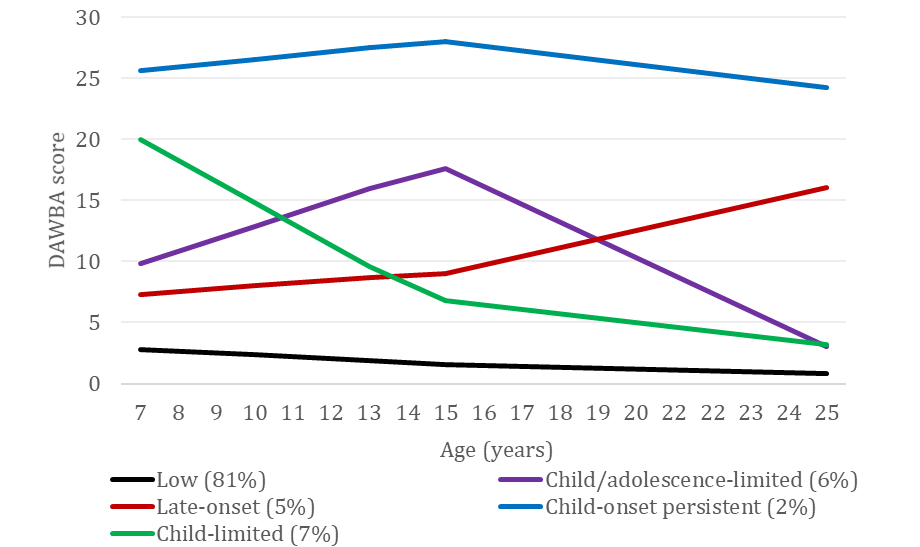 |

| **Supplementary** **Figure 4d-f.** Development and Well-Being Assessment score by class |
| --- |
| d) 5 data-points (complete cases: N=2,364): 3-class solution |
| 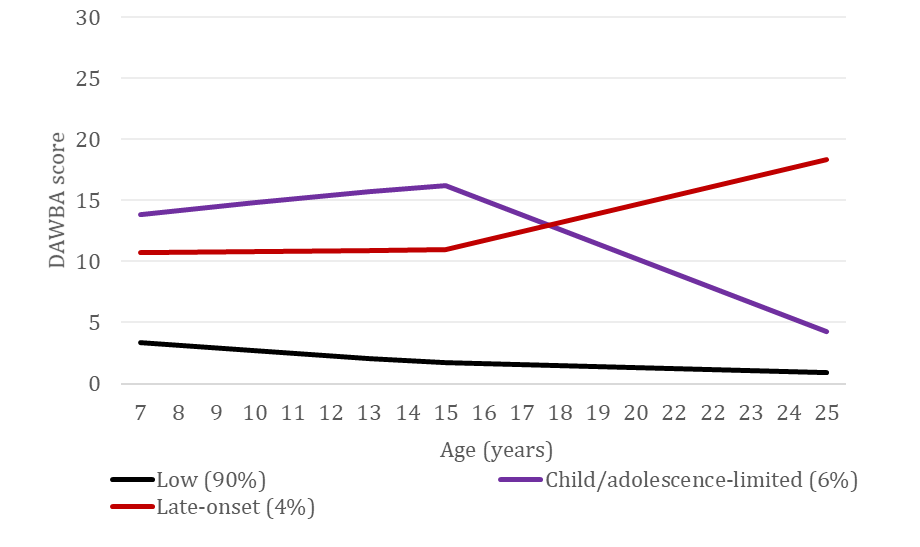 |
| e) 5 data-points (complete cases: N=2,364): 4-class solution |
| 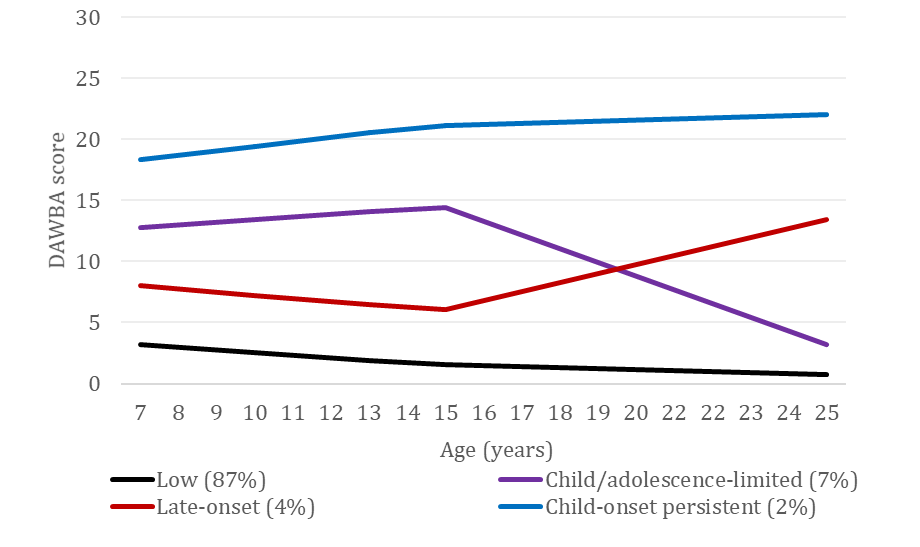 |
| f) 5 data-points (complete cases: N=2,364): 5-class solution |
| 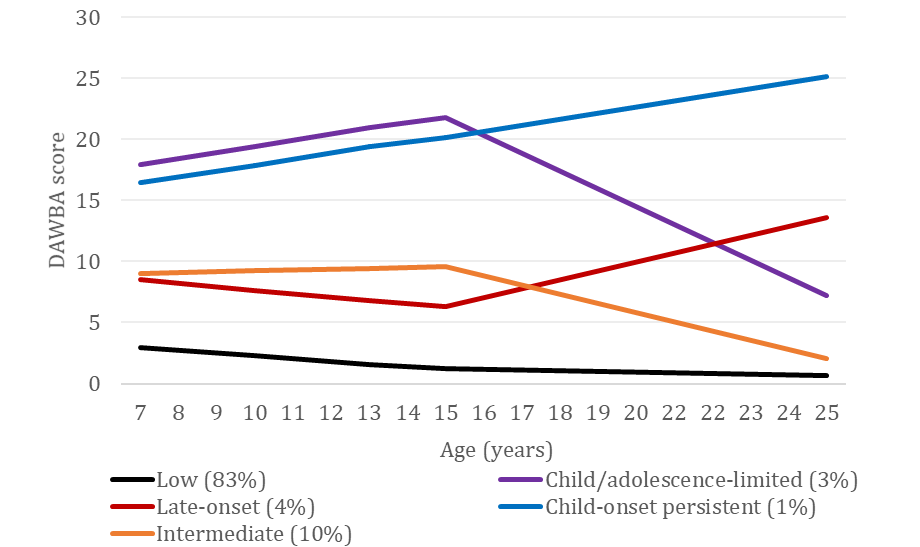 |
